# Predicting the functions of a protein from its ability to associate with other molecules

**DOI:** 10.1186/s12859-016-0882-3

**Published:** 2016-01-15

**Authors:** Kamal Taha, Paul D. Yoo

**Affiliations:** Department of Electrical and Computer Engineering, Khalifa University, Abu Dhabi, United Arab Emirates; Faculty of Science and Technology, Bournemouth University, Bournemouth, UK

## Abstract

**Background:**

All proteins associate with other molecules. These associated molecules are highly predictive of the potential functions of proteins. The association of a protein and a molecule can be determined from their co-occurrences in biomedical abstracts. Extensive semantically related co-occurrences of a protein’s name and a molecule’s name in the sentences of biomedical abstracts can be considered as indicative of the association between the protein and the molecule. Dependency parsers extract textual relations from a text by determining the grammatical relations between words in a sentence. They can be used for determining the textual relations between proteins and molecules. Despite their success, they may extract textual relations with low precision. This is because they do not consider the semantic relationships between terms in a sentence (i.e., they consider only the structural relationships between the terms). Moreover, they may not be well suited for complex sentences and for long-distance textual relations.

**Results:**

We introduce an information extraction system called PPFBM that predicts the functions of unannotated proteins from the molecules that associate with these proteins. PPFBM represents each protein by the other molecules that associate with it in the abstracts referenced in the protein’s entries in reliable biological databases. It automatically extracts each co-occurrence of a protein-molecule pair that represents *semantic relationship* between the pair. Towards this, we present novel semantic rules that identify the semantic relationship between each co-occurrence of a protein-molecule pair using the syntactic structures of sentences and linguistics theories. PPFBM determines the functions of an un-annotated protein *p* as follows. First, it determines the set *S*_*r*_ of annotated proteins that is semantically similar to *p* by matching the molecules representing *p* and the annotated proteins. Then, it assigns *p* the functional category *FC* if the significance of the frequency of occurrences of *S*_*r*_ in abstracts associated with proteins annotated with *FC* is statistically significantly different than the significance of the frequency of occurrences of *S*_*r*_ in abstracts associated with proteins annotated with all other functional categories. We evaluated the quality of PPFBM by comparing it experimentally with two other systems. Results showed marked improvement.

**Conclusions:**

The experimental results demonstrated that PPFBM outperforms other systems that predict protein function from the textual information found within biomedical abstracts. This is because these system do not consider the semantic relationships between terms in a sentence (i.e., they consider only the structural relationships between the terms). PPFBM’s performance over these system increases steadily as the number of training protein increases. That is, PPFBM’s prediction performance becomes more accurate constantly, as the size of training proteins gets larger. This is because every time a new set of test proteins is added to the current set of training proteins. A demo of PPFBM that annotates each input Yeast protein (SGD (Saccharomyces Genome Database). Available at: http://www.yeastgenome.org/download-data/curation) with the functions of Gene Ontology terms is available at: *(see Appendix for more details about the demo)*http://ecesrvr.kustar.ac.ae:8080/PPFBM/.

## Background

The advancement of genome sequencing techniques and the recent high-throughput technologies that study molecular mechanisms have led to exponential explosion of biomedical literatures. Fortunately, this rapid growing of biomedical literature has triggered an advancement in biological Natural Language processing (NLP) techniques that automatically extract useful information from the literature [1–3]. Information extraction aims at the automatic transferring of unstructured textual information into a structured form. Numerous NLP parsers have been widely used by the computational linguistics community, and have been employed to parse molecular biology data [3–15]. From these, the most popular ones are Bikel parser [5], the Collins parser [6], the Stanford parser [11, 16], Charniak parser [7], Berkeley Parser [17], Enju and Mogura Parsers [18], and Charniak-Lease parser [13]. These parsers fall under two categories: constituency and dependency [8, 19].

Constituency parsers performs syntactic analysis in a tree representation of the constituents constituting a sentence and the hierarchy that governs the associations among the constituents. These parsers analyze the structural relationships among constituents in each raw of input corpuses. In constituency parsing, lexical semantics analyze the meaning in the granularity of words, stems, suffixes, and prefixes [20]. Dependency parsers extract textual relations from a text by determining the grammatical relations between words in a sentence. Despite the success of most constituency and dependency parsers, they may extract textual relations with low precision. This is because they do not consider the semantic relationships between terms in a sentence (i.e., they consider only the structural relationships between the terms). Moreover, they may not be well suited for complex sentences and for long-distance textual relations.

A number of systems and approaches that employ NLP parsers have been proposed to parse biomedical texts to infer useful information such protein function and protein-protein interactions. The following is a survey of some of these popular systems. In GOstruct [21, 22], a protein *p* is annotated with functional category of a Gene Ontology (GO) term *t*, if *p* and *t* co-occur frequently in close proximity in PubMed abstracts. The abstracts were fed into a NLP pipeline, where abstracts are split into sentences, protein names are identified using BioNLP UIMA resources [23]. Text-KNN [24] represents a protein by the characteristic terms (i.e., GO terms) found within the biomedical abstracts associated with it. It annotates an un-annotated protein *p* with the functional categories of proteins represented by characteristic terms similar to *p*, using a k-nearest neighbor classifier. GOSTRUCT [25, 26] presents a system that aims to identify semantic associations among residues and proteins, using dependency graphs. GOSTRUCT can predict protein function from the protein sites mentioned in biomedical abstracts. It categorizes protein sites based on their protein structures determined by the amino acid residues found in biomedical abstracts.

We propose in this paper an information extraction system called PPFBM (**P**redicating **P**roteins **F**unctions from their **B**inding to other **M**olecules). PPFBM overcomes the limitations of most current constituency and dependency parsers outlined above as follows. It employs novel NLP dependency parsing and information extraction techniques that identify the *semantic relationship* between each pair of terms in a sentence using novel semantic rules. Moreover, it applies novel model and linguistic computational techniques for extracting the semantic relationship from different structural forms of terms in the sentences of biological texts. That is, PPFBM aims at enhancing the state of the art of biological text mining.

PPFBM analyzes biomedical texts in order to discover *protein function* information that is difficult to retrieve. Knowledge of protein function is crucial to the identification of gene-disease associations, cellular pathways, and drug design [4, 24, 27–34]. Towards this, PPFBM represents each protein by the other molecules associated with it and are found within the biomedical abstracts associated with the protein. This is because the other molecules associate with a protein are highly predictive of the potential functions of the protein [35]. That is, these molecules that strongly associate with a protein are good characteristics and indicators of the functions of the protein. All proteins bind to other molecules and these bindings determine the biological properties of the proteins such as their functions [27].

Not all the co-occurrences of a protein’s name and a molecule’s name in sentences can be considered as indicative of the association between the protein and the molecule. Therefore, PPFBM automatically extracts from biomedical abstracts each co-occurrence of a protein-molecule pair that represents *semantic relationship* between the pair. Towards this, we present novel association discovery techniques (i.e., semantic rules) that identify the semantic relationship between each co-occurrence of a protein-molecule pair using the syntactic structures of sentences and linguistics theories. After extracting the set of molecules, whose occurrences in abstracts represent semantic relationships with a protein, PPFBM selects the subset that is dominant and highly predictive of the protein’s functions. It then represents the protein with the selected subset of dominant molecules in the form of textual features.

PPFBM determines the functions of un-annotated protein *p* as follows. First, it determines the set *S*_*r*_ of annotated proteins that is semantically similar to *p* by matching the dominant molecules representing *p* and the dominant molecules representing the annotated proteins. Then, it determines the relative significance of the frequency of occurrences of set *S*_*r*_ in each abstract associated with a protein annotated with a functional category. Let *S*_*FC*_ be the significance of the frequency of occurrences of set *S*_*r*_ in biomedical abstracts associated with proteins annotated with the functional category *FC*. Let ${S}_{FC}^{\prime }$ be the significance of the frequency of occurrences of set *S*_*r*_ in biomedical abstracts associated with proteins annotated with all other functional categories. PPFBM will assign the un-annotated protein *p* the functional category *FC*, if *S*_*FC*_ is statistically significantly different than ${S}_{FC}^{\prime }$.

PPFBM locates and identifies the associations that describe semantic relationships between a protein and a molecule co-occurrences using novel dependency parsing and information extraction techniques. These techniques rely, in part, on empirically determined syntactic structures of sentences and linguistics theories. We present semantic search and information retrieval mechanisms to efficiently explore the associations that exist between protein-molecule pairs in the large amount of biomedical literature associated with proteins.

A demo of PPFBM that annotates each input Yeast protein [36] with the functions of Gene Ontology terms is available at: *(see*Appendix*for details)*http://ecesrvr.kustar.ac.ae:8080/PPFBM/

## Methods

### Representing a protein by a vector of weights

#### Extracting the molecules that associate with annotated training proteins from biological abstracts

We select a set of annotated proteins from a reliable biological database such as UniProtKB/Swiss-Prot [28]. The selected set will be used as a training protein dataset for PPFBM. The entry of each training protein in the biological database should have at least one reference to a PubMed abstract. We then retrieve the PubMed abstracts associated with the training proteins and referenced in the entry of the biological database. PPFBM extracts from these abstracts the molecules that associate with each of the selected training proteins. It automatically extracts from the retrieved abstracts each co-occurrence of a pair of protein and molecule that represents semantic relationship between the pair. These molecules will be used as text features to represent the training proteins. Our objective is to represent the training proteins using molecules that are highly predictive of their potential functions [24].

PPFBM is built on top of both ABNER Biomedical Named Entity Recognizer [37, 38] and ChEBI (Chemical Entities of Biological Interest) ontology [39]. ChEBI is a manually curated database and ontology that organizes small molecule knowledge [39]. PPFBM access a single ChEBI ontology file to determine ChEBI identifiers/terms. A list of ChEBI identifiers corresponds to small molecules at the leaf level of the ChEBI structural hierarchy. Then, ABNER is used for the identification of relevant named entities in biomedical texts that correspond to the ChEBI terms. Molecules are classified into five classes, RNA, protein, DNA, cell-type, and cell-line. The Co-reference Resolution connects occurrences of same proteins. Some of these occurrences are represented by terms such as “this protein”, “it”, “they”, etc. Also, lexical peculiarities in protein names (such as symbols and numbers) are identified. PPFBM employs a tokenizer and stemmer to align the sequence of words in a sentence and the names of molecules. A molecule’s stemmed words are aligned against abstracts. Finally, PPFBM performs a domain analysis to identify the related entities as well as the nature of their relationships.

#### Representing an annotated training protein by the other molecules that associate with it

Each protein *p* is represented by a *vector* of weights. That is, we view a protein *p* as a vector with one component corresponding to a molecule *m*_*i*_ that associate with *p*, together with a weight *w* (*m*_*i*_, *p*) on this component in the set of abstracts associated with *p*. The *w*(*m*_*i*_, *p*) represents the statistical significance of the co-occurrences of *m*_*i*_ and *p* based on their semantic relationships in the set of abstracts of PubMed associated with *p*. That is, *w*(*m*_*i*_, *p*) quantifies the likelihood of the association between *m*_*i*_ and *p* based on of their semantic relationship occurrences in the set of abstracts of PubMed associated with *p*. The co-occurrence of a molecule *m*_*i*_ and a protein *p* in a same sentence may not be necessary an indicative of the association between *m*_*i*_ and *p*. Therefore, the weight of the association between *m*_*i*_ and *p* pair relies, in part, on whether the co-occurrences of the pair are *semantically related*. That is, the weight ${w}_{A_j}\left({m}_i,\kern0.2em p\right)$ is based, in part, on whether the co-occurrences of the pair in abstract *A*_*j*_ are semantically related. A molecule that does not occur in abstracts, its weight is zero. Let ${w}_{A_j}\left({m}_i,\kern0.2em p\right)$ be the weight of the co-occurrences of *m*_*i*_ and *p* based on their semantic relationships in an abstract *A*_*j*_.

1 $$ w\left({m}_i,\kern0.3em p\right)=\frac{{\displaystyle \sum_{j=1}^{\left|A\right|}{w}_{A_j}\left({m}_i,\kern0.2em p\right)}}{\left|A\right|} $$

The weight ${w}_{A_j}\left({m}_i,\kern0.2em p\right)$ is calculated as shown in eq. 1.

2 $$ {w}_{A_j}\left({m}_i,\kern0.2em p\right)={T}_{A_j}\left({m}_i,\kern0.2em p\right)-{T}_{A_j}^{\prime}\left({m}_i,\kern0.2em p\right) $$

As shown in Table 1, let: (1) *o*_11_ and *o*_12_ be the observed frequencies of the co-occurrences of semantically related *m*_*i*_ and *p* pair in abstract *A*_*j*_, (2) *o*_21_ and *o*_22_ be the observed frequencies of the co-occurrences of semantically unrelated *m*_*i*_ and *p* pair in abstract *A*_*j*_, (3) *e*_11_ and *e*_12_ be the theoretical frequencies of the co-occurrences of semantically related *m*_*i*_ and *p* pair in abstract *A*_*j*_, and (4) *e*_21_ and *e*_22_ be the theoretical frequencies of the co-occurrences of semantically unrelated *m*_*i*_ and *p* pair in abstract *A*_*j*_. The operands ${T}_{A_j}\left({m}_i,\kern0.2em p\right)$ and ${T}_{A_j}^{\prime}\left({m}_i,\kern0.2em p\right)$ in Eq. 1 are calculated as follows:

**Table 1:**
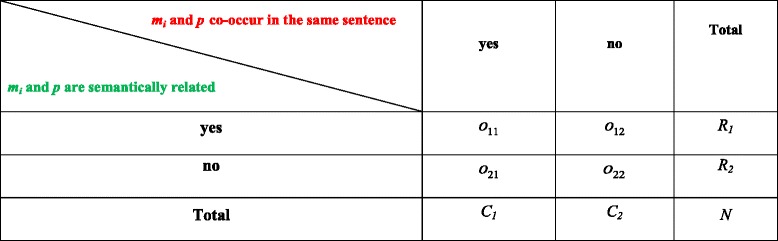
The distribution of semantically related and semantically unrelated co-occurrences of molecule *m*_*i*_ and Protein *p* Pair in an Abstract *A*_*j*_

- ➢ ${T}_{A_j}\left({m}_i,\kern0.2em p\right)$ is computed by normalizing the sum of the squared deviations of the observed frequencies *o*_11_ and *o*_12_ from the theoretical frequencies *e*_11_ and *e*_12_ in an abstract *A*_*j*_, where *m*_*i*_ and *p* may or may not co-occur in the same sentence. Thus, ${T}_{A_j}\left({m}_i,\kern0.2em p\right)$ is computed as follows: ${T}_{A_j}\left({m}_i,\kern0.2em p\right)=\kern0.4em \left(\frac{{\left({o}_{11}-{e}_{11}\right)}^2}{e_{11}}+\frac{{\left({o}_{12}-\kern0.3em {e}_{12}\right)}^2}{e_{12}}\right)$.

*If m*_*i*_*occurs in a different sentence than p, m*_*i*_* and p can be semantically related, if the two sentences are connected by a sentence connector (such as “moreover”, “however”, “otherwise”, “therefore”, etc.). In this case, the two sentences are represented by one common Part Of Sentence Tree* [40] *with one root node.*

- ➢ ${T}_{A_j}^{\prime}\left({m}_i,\kern0.2em p\right)$ is computed by normalizing the sum of the squared deviations of the observed frequencies *o*_21_ and *o*_22_ from the theoretical frequencies *e*_11_ and *e*_12_ in an abstract *A*_*j*_, where *m*_*i*_ and *p* may or may not co-occur in the same sentence. Thus, ${T}_{A_j}^{\prime}\left({m}_i,\kern0.2em p\right)$ is computed as follows: ${T}_{A_j}^{\prime}\left({m}_i,\kern0.2em p\right)=\kern0.8em \left(\kern0.1em \frac{{\left({o}_{21}-{e}_{21}\right)}^2}{e_{21}}+\frac{{\left({o}_{22}-{e}_{22}\right)}^2}{e_{22}}\right)$
- ➢ ${e}_{ixy}=\frac{R_x\times \kern0.4em {C}_y}{N}$. *N*: overall observed frequencies.

Each value of *o*_*xy*_ in Table 1 is computed using Eq. 2, where: (1) ${f}_{m_i,\kern0.1em p}$ denotes the frequency of co-occurrences of *m*_*i*_ and *p* pair that is semantically related *(in the case of o*_11_*and o*_12_*)* or semantically unrelated *(in the case of o*_21_*and o*_22_*)* in abstract *A*_*j*_, and (2) ${f}_{A_{m_i,\kern0.1em p}}$ denotes the frequency of abstracts containing co-occurrences of *m*_*i*_ and *p* pair that is semantically related *(in the case of o*_11_*and o*_12_*)* or semantically unrelated *(in the case of o*_21_*and o*_22_*)* in abstract *A*_*j*_. Equation 3 gives a value to *o*_*xy*_ based on the following factors:

1. It gives a high value to *o*_*xy*_ when *m*_*i*_ and *p* pair occurs many times within a small number of abstracts. This leads to discriminating power to those abstracts.
2. It gives a small value to *o*_*xy*_ when *m*_*i*_ and *p* pair occurs fewer times within a large number of abstracts.

3 $$ {o}_{x,\kern0.2em y}=\left\{\begin{array}{l}{f}_{m_i,\kern0.1em p}\times {f}_{A_{m_i,\kern0.1em p}}\kern0.5em \mathrm{if}\kern0.3em \mathrm{the}\kern0.3em \mathrm{pair}\kern0.5em \mathrm{occurs}\kern0.5em \mathrm{in}\kern0.3em \mathrm{at}\ \mathrm{least}\kern0.5em \mathrm{one}\kern0.5em \mathrm{abstract}\kern0.5em \\ {}0\kern0.8em \mathrm{otherwise}\end{array}\right. $$

where:

- $$ {f}_{m_i,\kern0.1em p}\kern0.4em =\left\{\begin{array}{l}\Big(c+\left(1-c\right)\kern0.2em \frac{n_{m_i,\kern0.1em p}}{ \max \left|{n}_{m_i,\kern0.1em p}\right|}\kern1.1em \left(\mathrm{see}\ \mathrm{note}\ 1\right)\\ {}1+\kern0.3em  \log \kern0.3em {n}_{m_i,\kern0.1em p}\kern0.72em \left(\mathrm{see}\ \mathrm{note}\ 2\right)\\ {}\frac{1}{A_{m_i,\kern0.1em p}}\kern1em \left(\mathrm{see}\ \mathrm{note}\ 3\right)\end{array}\right. $$
- $$ {f}_{A_{m_i,\kern0.1em p}}\kern0.5em =\left\{\begin{array}{l} \log \kern0.1em \left(1\kern0.3em +\frac{A_{m_i,\kern0.1em p}}{\left|A\right|}\right)\kern1.75em \left(\mathrm{see}\ \mathrm{note}\ 4\right)\\ {} \log \kern0.1em \left(1\kern0.3em +\frac{A_{m_i,\kern0.1em p}}{ \max \left|{n}_{m_i,\kern0.1em p}\right|}\right)\kern1.12em \left(\mathrm{see}\ \mathrm{note}\ 5\right)\end{array}\right. $$
- ${n}_{m_i,\kern0.1em p}$: Number of co-occurrences of *m*_*i*_ and *p* pair that is semantically related *(in the case of o*_11_*and o*_12_*)* or semantically unrelated *(in the case of o*_21_*and o*_22_*)* in abstract *A*_*j*_.
- $\max \left|{n}_{m_i,\kern0.1em p}\right|$: Number of co-occurrences of *m*_*i*_ and *p* pair that is semantically related *(in the case of o*_11_*and o*_12_*)* or semantically unrelated *(in the case of o*_21_*and o*_22_*)* in the abstract with the maximum frequency of the pair. This keeps the frequency multiplier of the pair from becoming greater than one.
- *c*: A constant ranges from zero to one.
- ${A}_{m_i,\kern0.1em p}$: Number of abstracts containing co-occurrences of *m*_*i*_ and *p* pair that is semantically related *(in the case of o*_11_*and o*_12_*)* or semantically unrelated *(in the case of o*_21_*and o*_22_*)*.
- |*A*|: Number of all abstracts in the database.

***Note 1:*** We use ${f}_{m_i,\kern0.1em p}=c+\left(1-c\right)\kern0.2em \frac{n_{m_i,\kern0.1em p}}{ \max \left|{n}_{m_i,\kern0.1em p}\right|}$, if we need to consider the order of appearance of *m*_*i*_ and *p* pairs in an abstract. This is important because, intuitively, the first appearances of the pair in an abstract should contribute more to the value of ${f}_{m_i,\kern0.1em p}$ than the subsequent appearances of the pair. In this equation, the first appearance of the pair in an abstract contributes much more than the remaining appearances. The constant 0 < *c* < 1 controls the balance between the initial and subsequent appearances of the pair. This method is preferred for use, if the abstracts are known to be associated with protein *p* (e.g., they are referenced in the protein’s entries in biological databases). This is because: (1) such abstracts usually contain occurrences of different molecules that associate with *p*, and (2) the molecules that appear first in the abstracts are usually more important to *p* (therefore, they should contribute more to the value of ${f}_{m_i,\kern0.1em p}$). This method may not be as effective for randomly selected abstracts, because some of the molecules that occur in these abstracts may not even associate/bind to *p*; therefore, ranking molecules based on their order of appearances in these abstracts is useless.

***Note 2:*** We use ${f}_{m_i,\kern0.1em p}=1+\kern0.3em  \log \kern0.3em {n}_{m_i,\kern0.1em p}$, if we need to: (1) give more diminishing returns as the co-occurrence frequency of *m*_*i*_ and *p* pair increases, and (2) have the co-occurrence of *m*_*i*_ and *p* pair to be very frequent in order for the frequency contribution value to be greater than four. The logarithm used in the formula gives diminishing returns as molecule frequencies increase. This method is preferred for use, if some of the abstracts are known to be associated with *p*, while the other ones are not. Intuitively, the frequencies of the molecules that associate with *p* in the former abstracts are much higher than the later ones. This may cause the contribution to the value ${f}_{m_i,\kern0.1em p}$ of the molecules in the later abstracts to be negligible. This method corrects this problem by giving diminishing returns as molecule frequencies in the former abstracts increase.

***Note 3:*** We use ${f}_{m_i,\kern0.1em p}=\frac{1}{A_{m_i,\kern0.1em p}}$, if we need to consider: (1) ${f}_{m_i,\kern0.1em p}$ as a local measure of the co-occurrences of *m*_*i*_ and *p* pair, and (2) a rank is a measure of importance. In this case, ${f}_{m_i,\kern0.1em p}$ is a global measure, invertly proportional to the number of abstracts containing the pair in the whole database. This method is preferred for use, if it is expected that the frequencies of molecules are sparsely distributed in the different abstracts (i.e., molecule frequencies are not dense in only some of the abstracts).

***Note 4:*** We use ${f}_{A_{m_i,\kern0.1em p}}= \log \kern0.1em \left(1\kern0.3em +\frac{\left|A\right|}{A_{m_i,\kern0.1em p}}\right)$, if we need to prevent a co-occurrence of *m*_*i*_ and *p* pair for which ${A}_{m_i,\kern0.1em p}= 1$ from being regarded as twice as important as another pair for which ${A}_{m_i,\kern0.1em p}$*= 2*. The logarithm included in the formula prevents a molecule for which ${A}_{m_i,\kern0.1em p}$*= l* from being regarded as twice as important as a molecule for which ${A}_{m_i,\kern0.1em p}$*= 2.* This method is preferred for use, if the abstracts have the same size or close sizes.

***Note 5:*** We use ${f}_{A_{m_i,\kern0.1em p}}= \log \kern0.1em \left(1\kern0.3em +\frac{ \max \left|{n}_{m_i,\kern0.1em p}\right|}{A_{m_i,\kern0.1em p}}\right)$, if we need to consider only the abstracts that contain co-occurrences of *m*_*i*_ and *p* pair for computing the value of ${f}_{A_{m_i,\kern0.1em p}}$ (i.e., if we want to disregard abstracts that do not contain co-occurrences of the pair).

**Table:** 

*Running Example:*
We illustrate some of the concepts presented in this paper using a running example pertaining to protein PA1535. We illustrate in the running example how the molecules associated with PA1535 can be used as a vector of weights to represent the protein. In Example 1, we present the abstract of Förster et al. [41] and describe how the weight of the co-occurrences of each molecule and protein PA1535 is computed based on their semantic relationships in the abstract. In Example 2, we illustrate how the weights of associations between 10 molecules and protein PA1535 are computed based on their co-occurrences in 12 abstracts associated with protein PA1535. We retrieved the 12 PubMed abstracts associated with protein PA1535 and referenced in the entry of UniProtKB/Swiss-Prot [28]. In Example 3, we illustrate how the beats/looses scores and normalized weights of the 10 molecules that associate with PA1535 are computed based on their co-occurrences in the 12 Abstracts.

*Example 1:* In this example, we describe how ${w}_{A_j}\left({m}_i,\kern0.2em p\right)$ in Eq. 2 is computed for protein PA1535. We selected the abstract of the paper Förster et al. [41] as *A*_*j*_ (Förster et al. is one of the 12 papers associated with protein PA1535). We describe how the weight of associations between molecules and protein PA1535 are computed based on their semantic relationships in the abstract of Förster et al. That is, we describe how ${w}_{A_{\mathrm{F}\ddot{\mathrm{o}} \mathrm{rster}\ \mathrm{e}\mathrm{t}\ \mathrm{al}.2008}}\left({m}_i,\kern0.2em PA1535\right)$ is computed. The abstract of Förster et al. [41] is shown below:

“*The atuRABCDEFGH gene cluster is essential for acyclic terpene utilization (Atu) in Pseudomonas aeruginosa. The biochemical functions of most Atu proteins have not been experimentally verified; exceptions are AtuC/AtuF, which constitute the two subunits of geranyl-CoA carboxylase, the key enzyme of the Atu pathway. In this study we investigated the biochemical function of AtuD and of the PA1535 gene product, a protein related to AtuD in amino acid sequence. 2D gel electrophoresis showed that AtuD and the PA1535 protein were specifically expressed in cells grown on acyclic terpenes but were absent in isovalerate- or succinate-grown cells. Mutant analysis indicated that AtuD but not the product of PA1535 is essential for acyclic terpene utilization. AtuD and PA1535 gene product were expressed in recombinant Escherichia coli and purified to homogeneity. Purified AtuD showed citronellyl-CoA dehydrogenase activity and high affinity to citronellyl-CoA. AtuD was inactive with octanoyl-CoA, 5-methylhex-4-enoyl-CoA or isovaleryl-CoA. Purified PA1535 gene product revealed high citronellyl-CoA dehydrogenase activity but had significantly lower affinity than AtuD to citronellyl-CoA. Purified PA1535 protein additionally utilized octanoyl-CoA as substrate. To our knowledge AtuD is the first acyl-CoA dehydrogenase with a documented substrate specificity for terpenoid molecule structure and is essential for a functional Atu pathway. Potential other terpenoid-CoA dehydrogenases were found in the genomes of Pseudomonas citronellolis, Marinobacter aquaeolei and Hahella chejuensis but were absent in non-acyclic terpene-utilizing bacteria*”.

Table 2 shows how ${w}_{A_{\mathrm{F}\ddot{\mathrm{o}} \mathrm{rster}\ \mathrm{e}\mathrm{t}\ \mathrm{al}.2008}}\left({m}_i,\kern0.2em PA1535\right)$ is computed using Eq. 2, where ${n}_{m_i,\kern0.1em p}$ is the number of co-occurrences of *m*_*i*_ and PA1535 pairs in the abstract of Förster et al. [41].

**Table 2:** The weight of associations between 10 molecules and protein PA1535 based on their co-occurrences in the abstract of Förster et al. [41]

*Molecule*	*n*_*mi, p*_		*A*_*mi, p*_		*f*_*mi, p*_		*f*_*Ami, p*_		*T*_*Aj*_ *(m*_*i*_ *, p)*	*T'*_*Aj*_ *(m*_*i*_ *, p)*	*w*_*Förster*_ *(m*_*i*_ *, p)*
	related	unrelated	related	unrelated	related	unrelated	related	unrelated			
AtuD	4	3	11	9	1.6	1.5	0.24	0.18	0.069	0.049	0.020
citronellyl-CoA	2	2	12	8	1.3	1.3	0.22	0.19	0.116	0.099	0.017
octanoyl-CoA	2	1	10	9	1.3	1	0.25	0.23	0.115	0.103	0.012
terpenoid-CoA	1	1	10	8	1	1	0.23	0.26	0.009	0.001	0.008
isovaleryl-CoA	1	2	2	6	1	1.3	0.12	0.22	0.005	0.002	0.003
Docosenoyl-CoA	0	0	9	7	0	0	0.24	0.25	0	0	0
OPC4-CoA	0	0	11	6	0	0	0.24	0.24	0	0	0
Sirodesmin H	0	0	5	8	0	0	0.21	0.28	0	0	0
OPC8-CoA	0	0	7	3	0	0	0.23	0.18	0	0	0
3-dipole	0	0	4	2	0	0	0.22	0.15	0	0	0

*Example 2:* Table 3 shows the weight of associations between 10 molecules and protein PA1535 based on their co-occurrences in 12 abstracts associated with the protein. Each cell in the table shows the weight of co-occurrences of *m*_*i*_ and *p* based on their semantic relationships in abstract *A*_*j*_ (i.e., ${w}_{A_j}\left({m}_i,\kern0.2em p\right)$)

**Table 3:** The weight of associations between 10 molecules and protein PA1535 based on their co-occurrences in 12 abstracts

molecule	*AtuD*	*citronellyl-CoA*	*octanoyl-CoA*	*terpenoid-CoA*	*isovaleryl-CoA*	Docosenoyl-CoA	OPC4-CoA	Sirodesmin H	OPC8-CoA	3-dipole
Abstract										
A_1_	0.020	0.017	0.012	0.008	0.003	0	0	0	0	0
A_2_	0.060	_0_	_0_	_0_	0.778	_0_	0.060	0.270	0.060	_0_
A_3_	_0_	0.060	0.778	0.060	_0_	_0_	_0_	0.060	_0_	0.088
A_4_	0.060	0.060	0.118	_0_	_0_	0.270	_0_	_0_	0.088	_0_
A_5_	0.060	_0_	_0_	_0_	0.778	_0_	0.060	0.270	0.060	_0_
A_6_	0	0.652	0	0.055	0.121	0	0.004	0	0	0.058
A_7_	0.493	0.116	0	0.008	0.072	0.002	0	0.603	0	0
A_8_	0	0	0.387	0.184	0	0	0.035	0	0.004	0.002
A_9_	0	0.002	0.0548	0	0.735	0.017	0	0.357	0	0.085
A_10_	0.664	0.183	0	0.006	0	0	0.736	0	0.002	0.006
A_11_	0.068	0.389	0.216	0.003	0	0.047	0.009	0	0	0.364
A_12_	0.213	0	0.735	0	0.043	0.003	0	0.007	0	0

#### Representing an annotated training protein by only the dominant molecules that associate with it

A molecule could be uninformative, if it has only few occurrences in abstracts and/or is assigned a high weight even though it is found in abstracts associated with many other protein classes. Some of these abstracts may contain only a few occurrences of a molecule associated with many proteins annotated with different functional classes. Including uninformative molecules could lead to misclassifying proteins of small function classes into the larger classes and vice versa. To overcome this problem, we should refine the set of molecules representing a protein by excluding the uninformative molecules and keeping only the dominant ones (i.e., the ones that have frequent occurrences in abstracts that are not associated with many other protein classes).

Towards this, we assign a score to each molecule *m* representing a protein *p*. The score reflects the dominance status of *m* relative to the other molecules representing *p*. First, we determine the pairwise *beats* and *looses* for each molecule contained in the abstracts associated with the protein *p*. Molecule *m*_*i*_ beats molecule *m*_*j*_, if the number of times that the weights of *m*_*i*_ (e.g., Table 3) is greater than that of *m*_*j*_ in abstracts. Then, each molecule *m* is assigned a score, which is the difference between the number of times that *m* beats the other molecules and the number of times it loses in the abstracts.

***Definition 1 – A score of a molecule:****Let m*_*i*_ > *m*_*j*_*denote: the number of times that the weights of molecule m*_*i*_*is greater than that of m*_*j*_ in abstracts*. Let S(m*_*i*_*, p) denote the score of association between molecule m*_*i*_*and protein p. Given the dominance relation* > *on the set of molecules V*_*p*_*for protein p, the score S(m*_*i*_*, p) equals:* |{*m*_*j*_ ∈ *V*_*P*_ : *m*_*i*_ > *m*_*j*_}| − |{*m*_*j*_ ∈ *V*_*p*_ : *m*_*j*_ > *m*_*i*_}|

The following are some of the characteristics of the above scoring approach: (1) the overall sum of molecules’ scores is zero, and (2) the *highest possible* score is (*n*−1) and the *lowest possible* score is –(*n*−1), where *n* is the number of molecules. We also compute $\overline{w}\left({m}_i,\kern0.2em p\right)$, the normalized weight of association between molecule *m*_*i*_ and protein *p* in abstracts. We compute $\overline{w}\left({m}_i,\kern0.2em p\right)$ by summing the positive of the most negative score and each other score and then normalizing the resulting values. Consider for example Table 4. The most negative score is −9. The positive of the most negative score (i.e., + 9) is summed to each score, as follows: (9 + 6 = 15), (9 + 5 = 14), (9 + 8 = 17), (9 + 2 = 11), (9 + 3 = 12), (9 − 6 = 3), (9 − 5 = 4), (9 + 1 = 10), (9 − 9 = 0), and (9 − 1 = 8). Finally, the resulting values are normalized as shown in the last row in Table 4 (i.e., row $\overline{w}\left({m}_i,\kern0.1em p\right)$).

**Table 4:** Beats/looses scores and normalized weights of the 10 molecules that associate with protein PA1535 based on their co-occurrences in 12 abstracts, calculated based on their weights shown in Table 3

	AtuD	citronellyl-CoA	octanoyl-CoA	terpenoid-CoA	isovaleryl-CoA	Docosenoyl-CoA	OPC4-CoA	Sirodesmin H	OPC8-CoA	3-dipole
AtuD	0	-	+	-	-	-	-	0	-	-
citronellyl-CoA	_+_	_0_	_0_	_−_	_−_	_−_	_−_	_0_	_−_	_−_
octanoyl-CoA	_−_	_0_	_0_	_−_	_−_	_−_	_−_	_−_	_−_	_−_
terpenoid-CoA	_+_	_+_	_+_	_0_	_+_	_−_	_−_	_0_	_−_	_+_
isovaleryl-CoA	_+_	_+_	_+_	_−_	_0_	_−_	_−_	_−_	_−_	_−_
Docosenoyl-CoA	_+_	_+_	_+_	+	+	0	0	+	-	+
OPC4-CoA	_+_	_+_	_+_	+	+	0	0	+	-	0
Sirodesmin H	0	0	+	0	+	-	-	0	-	0
OPC8-CoA	_+_	_+_	_+_	+	+	+	+	+	0	+
3-dipole	+	+	+	-	+	-	0	0	-	0
*S*(*m*_*i*_, *p*)	+6	+5	+8	+2	+3	−6	−5	+1	−9	−1
$\overline{w}\left({m}_i,\kern0.1em p\right)$	0.16	0.15	0.18	0.12	0.13	0.03	0.04	0.10	0	0.09

The Symbol “+” denotes that molecule *m*_*i*_ (column) Beats molecule *m*_*j*_ (row) in the Abstracts, while “-” denotes that *m*_*i*_ Lost. “0” denotes that *m*_*i*_ and *m*_*j*_ have the same Number of Beats and Looses. *S(m*_*i*_ *, p)* and $\overline{w}\left({m}_i,\kern0.2em p\right)$ denote the Score and Normalized Weight, respectively, of Molecule *m*_*i*_ in The 12 Abstracts. An Entry is based on Column-Row Order

*Example 3:* Table 4 show the same 10 molecules presented in Example 2 and Table 3 after calculating the scores of their associations with protein *p* in the 12 abstracts. The Table illustrates how the score *S(m*_*i*_*, p)* and normalized weight $\overline{w}\left({m}_i,\kern0.2em p\right)$ of the associations between the 10 molecules and protein PA1535 are calculated based on the weights shown in Table 3. Consider for example Table 3. *AtuD* beat *citronellyl-CoA* in six abstracts, *citronellyl-CoA* beat *AtuD* in four abstracts, and the two molecules have the same weight in two abstracts. Therefore, the symbol “-“is placed in the entry (*citronellyl-CoA*, *AtuD*) of Table 4 to denote that *citronellyl-CoA* lost to *AtuD (an entry is based on column-row order)*.

Then, the molecules are ordered by their normalized weights. The molecules with the most normalized weights are considered the *dominant* molecules for the protein *p*. The remaining molecules will be considered uninformative and will be excluded from the inclusion within the set of molecules representing *p*. Thus, protein *p* will be represented by only the dominant molecules as described above. That is, each protein is represented by only the dominant molecules associated with it. From the set *V*_*p*_ of molecules associated with *p*, the subset *Ṽ*_*p*_ ⊂ *V*_*p*_ is considered the dominant ones for *p*, if every molecules ∈ *Ṽ*_*p*_ satisfies the following:

1. It dominates every molecule *m*′ ∈ *V*_*p*_, *m*′ ∉ *Ṽ*_*p*_, (i.e., the normalized weight of *m* is greater than the normalized weight of each *m*′).
2. It acquires a normalized weight $\overline{w}$ (*m*, *p*) greater than a threshold *β. β* is a value lower than the mean normalized weight by the standard error of the normalized mean.

4 $$ \beta =\kern0.6em \frac{1-\kern0.5em \sqrt{{\displaystyle \sum_{\forall {m}_j\in {V}_p}{\left(\overline{w}\left({m}_j,\kern0.2em p\right)-\frac{1}{\left|{V}_p\right|}\right)}^2}}}{\left|{V}_p\right|} $$

***Definition 2 – Dominant molecule:****Let V*_*p*_*be the set of molecules for a protein p. Let*$\overline{w}\left({m}_i,\kern0.2em p\right)$*be the normalized weight of a molecule m*_*i*_ ∈ *V*_*p*_ associated with *p. The subset Ṽ*_*p*_ ⊂ *V*_*p*_*of the dominant molecules for p with the maximal weights is given by: {m*_*i*_ ∈ *V*_*p*_*:*$\overline{w}\left({m}_i,\kern0.2em p\right)\ge \overline{w}\left({m}_j,\kern0.2em p\right)$*, for all m*_*j*_ ∈ *V*_*p*_*, and*$\overline{w}\left({m}_i,\kern0.2em p\right)>\beta$*}*

We model protein *p* as a vector *Ṽ*_*p*_, with one component corresponding to a molecule *m*_*i*_, together with $\overline{w}$ (*m*_*i*_, *p*) on this component. Thus, *Ṽ*_*p*_ = {(*m*_*1*_, $\overline{w}$ (*m*_*1*_, *p*)), …, (*m*_*m*_, $\overline{w}$ (*m*_*m*_, *p*))}, where *m*_*i*_ is a dominant molecule in the set of abstracts associated with protein *p*.

#### Determining whether an annotated protein and a molecule are semantically related in a sentence

The co-occurrence of a molecule *m*_*i*_ and a protein *p* in the same sentence may not be an indicative of the association between *m*_*i*_ and *p*. Therefore, the weight of the association between *m*_*i*_ and *p* relies, in part, on whether the co-occurrences of the pair are *semantically related*. For example, the weights ${w}_{A_j}\left({m}_i,\kern0.2em p\right)$ in Table 3 are based, in part, on whether the co-occurrences of the 10 molecules and protein PA1535 in the 12 abstracts are *semantically related*. In this section, we propose semantic rules that determine whether a co-occurrence of a molecule and a protein in a sentence is semantically related. In each of the next subsections, we propose semantic rules based on linguistics theories and the syntactic structures of sentences.

In each of the next two subsections, we illustrate our proposed rules using sentences extracted from biomedical literature. In these examples, we show how the semantic relationships between molecules/proteins can be determined using our proposed rules. We divide each sentence into simple sentences using dependency grammar. Each simple sentence is an independent clause, which contains a subject and a predicate. We place each independent clause inside a rectangle for easy reference. In each example, the words that comprise a sentence are tagged as follows: (N) for noun, (V) for verb, (PREP) for preposition, and (PRON) for pronoun.

#### Sentences containing pronouns defining antecedents

According to linguistics, an antecedent noun is usually related to the subsequent noun(s), if the subsequent noun(s) is connected to the antecedent by a pronoun (such as “which”, “who”, “it”, “whom”, and “that”) [42]. We propose our first semantic rules based on this linguistic observation, as follows:

1. An antecedent noun is semantically related to a subsequent noun(s), if the two nouns are connected by a pronoun. Towards this, PPFBM replaces each pronoun with the *closest* noun found under the *predecessor independent clause*. This conforms to grammar and linguistics, which treat a pronoun as a word that can be substituted by a noun or noun phrase. In Examples 4–8, we strikethrough each pronoun and replace it with the *closest* noun found under the predecessor independent clause.
2. An explicit or implicit pronoun preceded by a conjunction (i.e., “and” and “or”) refers to the *subject* of closest predecessor independent clause. In Examples 4–8, we strikethrough each pronoun preceded by a conjunction and replace it with the *subject* of closest predecessor independent clause. In the case of an *implicit* pronoun preceded by a conjunction, we also replace it with the *subject* of closest predecessor independent clause.

For the sake of clarification, we perform the following in Examples 4–8:

1. We type the subject of the first independent clause using a different font.
2. We type each noun that replaces a pronoun: (1) in italics, (2) in a different font, and (3) place quotation marks around it. The replacement noun plays the role of the subject of the independent clause that comes immediately after the pronoun.

In Examples 4–8, we demonstrate how these semantic rules conform to the linguistics theory stated above. We determine the semantic relationships between each pair of molecules/proteins. Recall that all nouns (including the replacement nouns) within an independent clause are semantically related.

*Example 4:* Consider the following sentence: *“Coenzymes are the organic molecules Citronellyl-CoA and OPC4-CoA that bind to the active site of the GGPS1 protein”*. The following is the syntactic structure of the sentence in terms of its constituents of independent clauses.

The pronoun “that” is replaced by the *closest* noun(s) found under the predecessor independent clause (i.e., the nouns “Citronellyl-CoA” and “OPC4-CoA”), which become the subject nouns of the second independent clause. Therefore, the nouns “Citronellyl-CoA” and “OPC4-CoA” are semantically related to “GGPS1 protein”.

*Example 5:* Consider the sentence: *“It is cleaved to release* 53 amino-acid molecule, *which binds to the protein ADIPOR1 and interacts with the protein BMPR1A”*. The following is the syntactic structure of the sentence in terms of its constituents of independent clauses.

In the second independent clause, the pronoun “which” is replaced by the *closest* noun under the predecessor independent clause (i.e., the noun “53 amino-acid molecule”), which becomes the subject of the second independent clause. Therefore, “53 amino-acid molecule” and “protein ADIPOR1” are semantically related. In the third independent clause, the *implicit pronoun* that follows the conjunction “and” is replaced by the *subject* noun of the closest predecessor independent clause (i.e., the noun “53 amino-acid molecule”), which becomes the subject of the third independent clause. Therefore, the nouns “53 amino-acid molecule” and “protein BMPR1A” are semantically related.

*Example 6:* Consider the following sentence: *“Protein MshD acetyltransferase is composed of two GNAT domains, and it binds molecule AcCoA”*. The following is the syntactic structure of the sentence in terms of its constituents of independent clauses.

Since the pronoun “it” follows the conjunction “and”, it is replaced by the *subject* noun of the closest predecessor independent clause (i.e., the noun “Protein MshD acetyltransferase”), which becomes the subject of the second independent clause. Therefore, “Protein MshD acetyltransferase” and “molecule of AcCoA” are semantically related.

*Example 7:* Consider the following sentence: *“Molecule acetyl CoA is a purified recombinant and it catalyzes the hydration* of *the yeast protein mak3”*. The following is the syntactic structure of the sentence in terms of its constituents of independent clauses.

Since the pronoun “it” follows the conjunction “and”, it is replaced by the *subject* noun of the closest predecessor independent clause (i.e., the noun “acetyl CoA”), which becomes the subject of the second independent clause. Therefore, molecule “acetyl CoA” and yeast protein “mak3” are semantically related.

*Example 8:* Consider the following sentence: *“Fkh2p binds cooperatively with Mcm1p, which interacts with the* Sid2p, *which interacts with Blt1p and binds to mob1p”*. The following is the syntactic structure of the sentence in terms of its constituents of independent clauses.

The subject protein “Fkh2p” is semantically related to the molecule protein “Mcm1p”. In the second independent clause, the pronoun “which” is replaced by the *closest* noun under the predecessor independent clause (i.e., the noun “Mcm1p”), which becomes the subject of the second independent clause. Therefore, the molecule proteins “Mcm1p” and “Sid2p” are semantically related. In the third independent clause, the pronoun “which” is replaced by the *closest* noun under the predecessor independent clause (i.e., the noun “Sid2p”), which becomes the subject of the third independent clause. Therefore, the molecule proteins “Sid2p” and “*Blt1*” are semantically related. In the fourth independent clause, the *implicit pronoun* that follows the conjunction “and” is replaced by the *subject* noun of the closest predecessor independent clause (i.e., the noun “Sid2p”), which becomes the subject of the fourth independent clause. Therefore, the molecule proteins “Sid2p” and “mob1p” are semantically related.

#### Sentences containing preposition modifiers

Our second proposed semantic rules are based on the following linguistics observations [43, 44]: (1) two independent clauses connected by a preposition modifier (such as “but”, “while”, and “whereas”) are usually unrelated, and (2) all nouns within an independent clause are usually related. The following are our proposed rules, which are based on the above observations:

1. The co-occurrence of a molecule and a protein pair in a sentence is considered semantically *unrelated*, if the two terms occur in two different independent clauses connected by a preposition modifier. This is because the two terms do not have dependency relationship in this case.
2. The co-occurrence of a molecule and a protein pair within an independent clause (i.e., inside a rectangle in our examples) is considered semantically *related*.

In Examples 9–11, we demonstrate how these semantic rules conform to the linguistics theory stated previously. In these sentences, we determine the semantic relationship between each pair of molecules/proteins in the sentences.

*Example 9:* Consider the sentence: *“Citronellyl-CoA and OPC4-CoA participate in the catalysis of GGPS1 but OPC8-CoA is a substrate of the reaction of OPCL1”*. Below is the syntactic structure of the sentence in terms of its constituents of independent clauses:

In the first independent clause, the organic molecules “Citronellyl-CoA” and “OPC4-CoA” are semantically related to the protein “GGPS1”. In the second independent clause, the molecule “OPC8-CoA” is semantically related to the protein “OPCL1”. However, each of “Citronellyl-CoA”, “OPC4-CoA”, and ‘GGPS1” is *unrelated* to each of “OPC8-CoA” and “OPCL1”, because they belong to two different independent clauses connected by the preposition modifier “but”.

*Example 10:* Consider the following sentence: “*The sequence of MshD is twice the length of GNAT and it binds CoASH and HSCoA, whereas ARL1 binds SCOCO and Golgin-245*”. The following is the syntactic structure of the sentence in terms of its constituents of independent clauses.

Since the pronoun “it” follows the conjunction “and”, it is replaced by the *subject* noun of the closest predecessor independent clause (i.e., the noun protein “MshD”), which becomes the subject of the second independent clause. Therefore, the protein “MshD” is semantically related to the molecules “CoASH” and “HSCoA”. In the third independent clause, the protein “ARL1” is semantically related to the molecules “SCOCO” and “Golgin-245”. However, each of “MshD”, “CoASH” and “HSCoA” is unrelated to each of “ARL1”, “SCOCO” and “Golgin-245”, because the first and second sets of nouns belong to two different independent clauses connected by the preposition modifier “whereas”.

*Example 11:* Consider the following sentence: *“caveolin-1 and caveolin-2 interact with c-src and Ha-ras, while cRAF-1 interacts with protein CDK4”*. Below is the syntactic structure of the sentence in terms of its constituents of independent clauses:

In the first independent clause, the proteins “caveolin-1”, “caveolin-2”, and “caveolin-3” are semantically related to the signalling molecules “c-src”, “Ha-ras”, and “GSa”. In the second independent clause, the proteins “cRAF-1” and “CDK4” are semantically related. However, each of “caveolin-1”, “caveolin-2”, “caveolin-3”, “c-src”, “Ha-ras”, and “GSa” is unrelated to each of “cRAF-1” and “CDK4”, because the two sets of nouns belong to two different independent clauses connected by the preposition modifier “while”.

### Determining the functions of an Un-annotated protein

#### Determining the semantic similarity between an Un-annotated protein and the Set of training proteins

Each annotated training protein *p* is represented by a vector *Ṽ*_*p*_ of the dominant molecules associated with *p* in biomedical abstracts. Let ${\tilde{V}}_{p^{\prime }}$ be the vector of weights representing an un-annotated protein *p*′. Each component in ${\tilde{V}}_{p^{\prime }}$ corresponds to a molecule *m*_*i*_ that associate with *p*′, together with a weight *w*(*m*_*i*_, *p*′) on this component. *w*(*m*_*i*_, *p*′) is determined from the reference works that describe the un-annotated protein *p*′ and is computed using the same techniques described in previously. Let *sim* ( *p*′, *p*) be the semantic similarity of *p*′ and an annotated training protein *p*, computed based on the similarity of ${\tilde{V}}_{p^{\prime }}$ and Ṽ_*p*_. PPFBM employs the cosine-based semantic similarity measure shown in Eq. 5 for measuring *sim* (*p*, *p*′). After measuring the semantic similarity of *p*′ and each annotated training protein *p*, we determine the set *S*_*r*_ of annotated training proteins that is semantically similar to *p*′.

5 $$ sim\kern0.4em \left(\kern0.1em {p}^{\prime },\kern0.2em p\kern0.1em \right)\kern0.4em =\kern0.3em \frac{{\displaystyle \sum_{\forall {m}_i\in \kern0.3em \left({\tilde{V}}_p\cap \kern0.3em {\tilde{V}}_{p^{\prime }}\right)}\kern0.3em \left(\left(\overline{w}\left({m}_i,\kern0.3em {p}^{\prime}\right)-\overline{\overline{w}}\left({m}_i,\kern0.3em {p}^{\prime}\right)\right)\kern0.6em \left(\overline{w}\left({m}_i,\kern0.3em p\right)-\kern0.3em \overline{\overline{w}}\left({m}_i,\kern0.3em p\right)\right)\right)}}{\sqrt{{\displaystyle \sum_{\forall {m}_i\in \kern0.3em \left({\tilde{V}}_p\cap \kern0.3em {\tilde{V}}_{p^{\prime }}\right)}\kern0.4em {\left(\overline{w}\left({m}_i,\kern0.3em {p}^{\prime}\right)-\kern0.6em \overline{\overline{w}}\left({m}_i,\kern0.3em {p}^{\prime}\right)\right)}^2}\kern0.5em }\sqrt{{\displaystyle \sum_{\forall {m}_i\in \kern0.3em \left({\tilde{V}}_p\cap \kern0.3em {\tilde{V}}_{p^{\prime }}\right)}\kern0.2em {\left(\overline{w}\left({m}_i,\kern0.3em p\right)-\kern0.6em \overline{\overline{w}}\left({m}_i,\kern0.3em p\right)\right)}^2}}} $$

- ▪ $\overline{w}\left({m}_i,\kern0.2em p\right)$: Normalized weight of the semantic relationship association between a molecule *m*_*i*_ and an annotated protein *p* in abstracts associated with *p*.
- ▪ $\overline{w}\left({m}_i,\kern0.2em {p}^{\prime}\right)$: Weight of the semantic relationship associations between a molecule *m*_*i*_ and the un-annotated protein *p*′ in the reference works that describe *p*′.
- ▪ *Ṽ*_*p*_: Set of the dominant molecules that have semantic relationship associations with *p* in biomedical abstracts.
- ▪ ${\tilde{V}}_{p^{\prime }}$_:_ Set of the molecules that have semantic relationship associations with *p*′ in the reference works describing *p*′.
- ▪ ${\tilde{V}}_p\cap \kern0.3em {\tilde{V}}_{p^{\prime }}$: Set of the molecules representing both *P* and *p*′.
- ▪ $\overline{\overline{w}}\left({m}_i,\kern0.2em p\right)$: Mean weight of the common molecules representing both *p* and *p*′ in the vector representing *p*, where: $\overline{\overline{w}}\left({m}_i,\kern0.2em p\right)=\frac{{\displaystyle \sum_{\forall {m}_i\in \kern0.3em \left({\tilde{V}}_p\cap \kern0.3em {\tilde{V}}_{p^{\prime }}\right)}\overline{w}\left({m}_i,\kern0.2em p\right)}}{\left|\kern0.2em {\tilde{V}}_p\cap \kern0.4em {\tilde{V}}_{p^{\prime }}\right|}$

- $\overline{\overline{w}}\Big({m}_i,\kern0.2em {p}^{\prime }$): Mean weight of the common molecules representing both *p* and *p*′ in the vector representing *p*′, where:

$$ \overline{\overline{w}}\left({m}_i,\kern0.2em {p}^{\prime}\right)=\frac{{\displaystyle \sum_{\forall {m}_i\in \kern0.3em \left({\tilde{V}}_p\cap \kern0.3em {\tilde{V}}_{p^{\prime }}\right)}\overline{w}\left({m}_i,\kern0.2em {p}^{\prime}\right)}}{\left|\kern0.2em {\tilde{V}}_p\cap \kern0.4em {\tilde{V}}_{p^{\prime }}\right|} $$

#### Determining the functional category of an Un-annotated protein

As described previously, we determine the set *S*_*r*_ of annotated training proteins that is semantically similar to the un-annotated protein *p*′ using Eq. 5. Let *S*_*FC*_ be the significance of the frequency of occurrences of set *S*_*r*_ in PubMed abstracts associated with proteins annotated with the functional category *FC*. Let ${S}_{FC}^{\prime }$ be the significance of the frequency of occurrences of set *S*_*r*_ in PubMed abstracts associated with proteins annotated with *all other* functional categories. The un-annotated protein *p*′ will be annotated with the functional category *FC*, if *S*_*FC*_ is statistically significantly different than *S*_*FC*_^′^. An abstract is determined to be associated with a protein, if it is referenced in the protein’s entry in a reliable biological database such as UniProtKB/Swiss-Prot [28].

PPFBM employs Z-score for determining the significance of the frequency of occurrences of set *S*_*r*_ in PubMed abstracts. That is, Z**-**score is used for determining the significance of the frequency of occurrences of *each* protein *p* ∈ *S*_*r*_ in *each* set of PubMed abstracts associated with proteins annotated with the same functional category. The Z**-**score for a protein *p* ∈ *S*_*r*_ in a set of PubMed abstracts associated with proteins annotated with a functional category *FC*, is the distance between the raw score for *p* and the population mean, as shown in Eq. 6:

6 $$ Z- score\kern0.3em =\kern0.3em \frac{\left(\frac{N_{FC}^p}{M_{FC}}\right)\kern0.6em -\kern0.7em \left(\frac{N_{F{C}^{\prime}}^p}{M_{F{C}^{\prime }}}\right)}{\sigma } $$

where:

- ➢ *N*_*FC*_^*p*^: Number of PubMed abstracts associated with proteins annotated with *FC* and contain occurrences of *p*.
- ➢ ${N}_{F{C}^{\prime}}^p$: Number of PubMed abstracts associated with proteins annotated with all other functional categories *FC*′ (i.e., *FC*′ ≠ *FC*) and contain occurrences of *p*.
- ➢ *M*_*FC*_: Overall number of PubMed abstracts associated with proteins annotated with *FC*.
- ➢ ${M}_{F{C}^{\prime }}$: Overall number of PubMed abstracts associated with proteins annotated with *FC*′.
- ➢ *σ*: Standard deviation of the population.

## Results and discussion

We implemented PPFBM in Java, run on Intel(R) Core(TM) i5-4200U processor, with a CPU of 2.30 GHz and RAM of 4 GB, under Windows 8. A demo of PPFBM that annotates each input Yeast protein [36] with the functions of Gene Ontology terms is available at: *(see*Appendix*for more details about the demo)*http://ecesrvr.kustar.ac.ae:8080/PPFBM/.

We experimentally evaluated the quality of PPFBM for predicting the functions of proteins by comparing it with GOstruct [21, 22] and Text-KNN [24]. The following are brief overviews of the two systems:

- ***GOstruct*** [21, 22]***:*** In the framework of GOstruct, a protein *p* is annotated with the functional category of a Gene Ontology (GO) term *t*, if *p* and concepts associated with *t* co-occur frequently in close proximity in PubMed abstracts. We re-implemented the framework of GOstruct exactly as described in [21, 22]. We also contacted some of the co-authors of the two papers to ensure accurate re-implementation of GOstruct. The following is a brief description of the methodology and tools used in the re-implementation. Abstracts are fed into a NLP pipeline, where they are split into sentences, and the co-mentions in these sentences are identified using BioNLP Apache Unstructured Information Management Architecture (UIMA) version 2.4 [23, 45]. UIMA creates a pipeline to automatically extract co-mentions of a specific protein and concepts associated with GO terms found within the abstracts. The version of UIMA we used employs LingPipe sentence-detector version 3.9.3 [46] to fragment text it into sentences. LingPipe is trained using Colorado Richly Annotated Full Text (CRAFT) corpus. Tokenization is done using PennBio tokenizer version 0.5 [47], which is distributed with ConceptMapper version August 2008 [48]. Protein names in abstracts are identified by mapping protein mentions to UniProt identifiers using a protein dictionary. GO terms and the concepts associated with them in abstracts are identified by looking up ConceptMapper dictionaries [49]. The co-mentions of a specific protein and concepts associated with GO terms are determined based on sentence spans. That is, co-mentions are mentions of a protein and concepts from the Gene Ontology that co-occur within a sentence. Each protein is represented by a vector. Each component of the vector represents the number of times that the protein co-occurs with concepts associated with a specific GO term. The GOstruct framework is available for download at: http://sourceforge.net/projects/strut/files/
- ***Text-KNN***[24]***:*** It represents a protein by the characteristic terms found within the biomedical abstracts associated with it. It annotates an un-annotated protein *p* with the functional categories of proteins represented by characteristic terms similar to *p*, using a k-nearest neighbour classifier.

We evaluate and compare the prediction accuracy of the three systems by measuring their performance for predicting the functions of each protein *P* in the dataset using the standard *Recall*, *Precision*, and *F-value* metrics shown below:

$$ \mathrm{Recall}=\kern0.3em \frac{c_p}{n_p},\kern0.5em \mathrm{Precision}=\kern0.3em \frac{c_p}{m_p},\kern0.5em F- value=\kern0.3em \frac{2\times \mathrm{Recall}\times \kern0.3em \mathrm{Precision}}{\mathrm{Recall}+\kern0.3em \mathrm{Precision}} $$

- *c*_*p*_: Number of *correctly* predicted functions for *P*.
- *n*_*p*_: Number of actual functions of *P*.
- *m*_*p*_: Number of predicted function for *P*.

### Compiling datasets for the evaluation

#### CAFA challenge dataset

We evaluated the systems using the Critical Assessment of Functional Annotation (CAFA) challenge dataset [24, 50]. The goal of the CAFA challenge is to evaluate automated protein function prediction algorithms. We used for the evaluation CAFA 2 (2013–2014) dataset. CAFA 2 challenge consisted originally of 100,816 un-annotated proteins at the time of submission deadline on January 20, 2014. By the 17th of February 2015, 26,643 of these proteins have become experimentally annotated and validated. Therefore, we did not follow the exact CAFA set up. Each of the selected proteins has been associated with at least one PubMed abstract according to its entry in UniProtKB database. We used for the evaluation the 26,643 proteins and the 94,846 PubMed abstracts associated with them according to their entries in UniProtKB database.

### Saccharomyces Genome Database (SGD)

We also evaluated the three systems using the complete 6086 Saccharomyces Genome Dataset (SGD) [36] as well as the 46,227 PubMed abstracts associated with the 6086 proteins according to their entries in UniProtKB database. SGD is a publicly available resource for the budding yeast *Saccharomyces cerevisiae*. SGD provides encyclopedic information about the yeast proteins, genome and its genes, and other encoded features. Experimental results on the functions and interactions of the yeast proteins are extracted by high-quality manual curation and are integrated within a well-developed database. This data is combined with high-throughput results. This combined collection of data is integrated with a variety of bioinformatics tools to help in experimental design and analysis and to allow discovery of new biological details. The SGD resource can be considered as a standard for functional description of budding yeast. It can also be considered as a platform from which to investigate related proteins and pathways. The SGD data is freely accessible to researchers and can be downloaded from [36].

### Gene ontology dataset

We also evaluated the three systems using Gene Ontology (GO) dataset [51]. The dataset consists of GO terms and the proteins annotated to the functions of these GO terms. We selected a fragment of GO graph containing 70 GO terms from the biological process sub-ontology. We also selected a fragment of GO graph containing 30 GO terms from the molecular function sub-ontology. Table 5 shows the number of proteins selected for the evaluations from these two sub-ontologies (i.e., 62,386 proteins annotated to the functions of GO terms from the biological process sub-ontology and 16,576 proteins annotated to the functions of GO terms from the molecular function sub-ontology). We downloaded the 100 GO terms and the 78,962 proteins annotated to their functions from [51]. We retrieved 577,486 PubMed abstracts associated with the 78,962 proteins based on the entries of these proteins in UniProtKB/Swiss-Prot database [28].

**Table 5:** The go dataset used in the experiments

	Biological process sub-ontology	Molecular function sub-ontology
No. of GO terms selected for the experiments	70	30
No. of proteins annotated to the GO terms	584, 973	604,625
No. of proteins selected for the experiments^a^	62,386	16,576

^a^We selected for the experiments only the proteins that: (1) are associated with at least one PubMed abstract based on their entries in UniProtKB [28], and (2) have experimental evidence code: IDA, IC, IPI, EXP, IEP, IMP, TAS, IC, or IGI

**Table 6:** Performance of predicting the biological process annotations using randomly selected sets of training and testing proteins

GO Term	Average depth (level) of GO term	Number of training proteins	Number of testing protein	PPFBM			GOstruct			Text-KNN		
				R	P	F	R	P	F	R	P	F
GO:0048856	4	2130	420	0.74	0.71	0.72	0.45	0.49	0.47	0.24	0.26	0.25
GO:0002009	4	633	125	0.55	0.60	0.57	0.37	0.35	0.36	0.19	0.22	0.20
GO:0072088	4	36	9	0.34	0.35	0.34	0.54	0.58	0.56	0.12	0.04	0.06
GO:0035295	4	1890	370	0.75	0.78	0.76	0.45	0.43	0.44	0.30	0.28	0.29
GO:0035239	4	1304	260	0.71	0.75	0.73	0.36	0.35	0.35	0.28	0.26	0.27
GO:0001763	4	865	173	0.66	0.65	0.65	0.43	0.45	0.44	0.20	0.24	0.22
GO:0072001	5	450	90	0.55	0.59	0.57	0.45	0.50	0.47	0.20	0.25	0.22
GO:0009653	5	1345	265	0.75	0.73	0.74	0.41	0.47	0.44	0.25	0.32	0.28
GO:0009888	5	859	171	0.66	0.67	0.66	0.35	0.39	0.37	0.18	0.23	0.20
GO:0048589	5	1828	360	0.76	0.8	0.78	0.54	0.57	0.55	0.25	0.27	0.26
GO:0060562	5	1212	240	0.71	0.74	0.72	0.43	0.47	0.45	0.23	0.27	0.25
GO:0001657	5	438	87	0.51	0.56	0.53	0.46	0.46	0.46	0.19	0.22	0.20
GO:0061138	5	792	158	0.69	0.75	0.72	0.42	0.45	0.43	0.15	0.21	0.18
GO:0060429	6	528	105	0.60	0.65	0.62	0.34	0.27	0.30	0.22	0.30	0.25
GO:0048731	6	1183	225	0.72	0.78	0.75	0.38	0.43	0.40	0.31	0.31	0.31
GO:0072009	6	86	20	0.38	0.41	0.39	0.45	0.49	0.47	0.09	0.07	0.08
GO:0001655	6	204	41	0.41	0.46	0.43	0.35	0.31	0.33	0.18	0.24	0.21
GO:0001822	6	110	30	0.39	0.48	0.43	0.37	0.41	0.39	0.19	0.15	0.17
GO:0072073	6	84	21	0.46	0.49	0.47	0.53	0.62	0.57	0.07	0.09	0.08
GO:0060560	6	1062	200	0.69	0.71	0.70	0.37	0.39	0.38	0.27	0.31	0.29
GO:0072033	6	61	13	0.29	0.33	0.31	0.48	0.55	0.51	0.09	0.2	0.12
GO:0060675	6	277	55	0.41	0.42	0.41	0.40	0.44	0.42	0.20	0.25	0.22
GO:0045165	6	1379	270	0.72	0.78	0.75	0.39	0.40	0.39	0.31	0.33	0.32
GO:0007267	6	1532	290	0.70	0.76	0.73	0.48	0.51	0.49	0.23	0.24	0.23
GO:0030154	6	1596	310	0.71	0.78	0.74	0.45	0.47	0.46	0.23	0.26	0.24
GO:0065008	6	1400	270	0.73	0.76	0.74	0.44	0.47	0.45	0.27	0.28	0.27
GO:0048754	6	687	137	0.55	0.57	0.56	0.37	0.39	0.38	0.17	0.20	0.18
GO:0009887	6	12	4	0.17	0.24	0.20	0.52	0.53	0.52	0.00	0.00	0.00
GO:0044699	6	1912	370	0.72	0.71	0.71	0.38	0.37	0.37	0.27	0.32	0.29
GO:2001141	6	1731	335	0.70	0.71	0.70	0.42	0.43	0.42	0.32	0.36	0.34
GO:0010468	6	1758	340	0.75	0.70	0.72	0.39	0.41	0.40	0.22	0.26	0.24
GO:2000112	6	1637	320	0.64	0.68	0.66	0.39	0.40	0.39	0.31	0.35	0.33
GO:0048513	7	1107	220	0.65	0.72	0.68	0.47	0.47	0.47	0.23	0.29	0.26
GO:0048729	7	465	93	0.55	0.62	0.58	0.39	0.40	0.39	0.19	0.23	0.21
GO:0001656	7	72	18	0.38	0.42	0.40	0.43	0.51	0.47	0.20	0.25	0.22
GO:0060993	7	109	21	0.39	0.42	0.40	0.42	0.43	0.42	0.16	0.23	0.19
GO:0072006	7	100	25	0.37	0.42	0.39	0.37	0.42	0.39	0.04	0.18	0.07
GO:0001658	7	402	80	0.52	0.57	0.54	0.44	0.45	0.44	0.22	0.25	0.23
GO:0061326	7	309	61	0.49	0.53	0.51	0.38	0.39	0.38	0.21	0.28	0.24
GO:0045168	7	459	91	0.79	0.82	0.80	0.43	0.45	0.44	0.23	0.23	0.23
GO:0051094	7	1768	340	0.75	0.81	0.78	0.49	0.52	0.50	0.31	0.35	0.33
GO:0051240	7	1780	340	0.76	0.79	0.77	0.44	0.44	0.44	0.28	0.32	0.30
GO:0022603	7	1850	350	0.67	0.70	0.68	0.39	0.41	0.40	0.33	0.35	0.34
GO:0072087	7	44	11	0.33	0.38	0.35	0.54	0.64	0.59	0.00	0.00	0.00
GO:0090183	7	345	69	0.50	0.56	0.53	0.43	0.42	0.42	0.22	0.29	0.25
G0:0061005	7	279	55	0.49	0.48	0.48	0.43	0.43	0.43	0.19	0.23	0.21
GO:0032835	7	338	67	0.45	0.53	0.49	0.40	0.42	0.41	0.20	0.26	0.23
GO:2000027	8	631	126	0.59	0.61	0.60	0.34	0.37	0.35	0.21	0.25	0.23
GO:0072080	8	241	48	0.40	0.43	0.41	0.36	0.36	0.36	0.21	0.23	0.22
GO:0003338	8	52	13	0.26	0.35	0.30	0.41	0.48	0.44	0.07	0.17	0.10
GO:0044767	8	1755	351	0.78	0.82	0.80	0.38	0.45	0.41	0.23	0.26	0.24
GO:0072028	8	48	12	0.36	0.38	0.37	0.42	0.54	0.47	0.00	0.00	0.00
GO:0006366	8	1840	350	0.67	0.71	0.69	0.45	0.47	0.46	0.24	0.27	0.25
GO:0006355	8	1804	350	0.51	0.55	0.53	0.38	0.39	0.38	0.30	0.29	0.29
GO:0031128	8	213	42	0.42	0.44	0.43	0.46	0.46	0.46	0.17	0.23	0.20
GO:0090184	8	1717	34	0.70	0.73	0.71	0.40	0.38	0.39	0.32	0.35	0.33
GO:0072210	8	72	18	0.39	0.46	0.42	0.45	0.46	0.45	0.00	0.00	0.00
GO:0072215	8	132	26	0.42	0.44	0.43	0.39	0.41	0.40	0.10	0.12	0.11
GO:0077273	8	199	39	0.46	0.47	0.46	0.39	0.42	0.40	0.15	0.17	0.16
GO:0072202	8	119	24	0.42	0.44	0.43	0.41	0.38	0.39	0.12	0.14	0.13
GO:0072207	8	125	25	0.41	0.45	0.43	0.33	0.41	0.37	0.09	0.12	0.10
GO:0072075	8	183	36	0.41	0.43	0.42	0.44	0.45	0.44	0.18	0.21	0.19
GO:0072170	8	108	28	0.32	0.36	0.34	0.39	0.42	0.40	0.11	0.15	0.13
GO:0072234	9	176	45	0.39	0.46	0.42	0.32	0.45	0.37	0.08	0.17	0.11
GO:0072017	9	104	20	0.38	0.47	0.42	0.40	0.45	0.42	0.15	0.19	0.17
GO:0072077	9	32	8	0.25	0.38	0.30	0.46	0.51	0.48	0.00	0.00	0.00
GO:0072078	9	148	38	0.36	0.39	0.37	0.40	0.41	0.40	0.14	0.16	0.15
GO:0072070	9	147	37	0.38	0.46	0.42	0.36	0.42	0.39	0.19	0.23	0.21
GO:0072050	9	67	15	0.38	0.45	0.41	0.40	0.38	0.39	0.14	0.07	0.09
GO:0006357	9	1992	390	0.69	0.75	0.72	0.39	0.45	0.42	0.31	0.32	0.31

The table shows the average depth (level) of each GO term in the biological process subontology and the accuracy of predicting the function of this term. R, P, and F DENOTE Recall, Precision, and F-value respectively

**Table 7:** Performance of predicting the molecular function annotations using randomly selected sets of training and testing proteins

GO Term	Average depth (level) of GO term	Number of training proteins	Number of testing proteins	PPFBM			GOstruct			Text-KNN		
				R	P	F	R	P	F	R	P	F
GO:0038023	4	830	210	0.62	0.67	0.55	0.41	0.43	0.53	0.25	0.33	0.28
GO:0009927	4	51	15	0.42	0.46	0.44	0.53	0.56	0.54	0.00	0.00	0.00
GO:0000156	4	1399	350	0.78	0.79	0.78	0.37	0.44	0.40	0.37	0.46	0.41
GO:0005057	4	1014	250	0.59	0.64	0.61	0.40	0.43	0.41	0.35	0.43	0.39
GO:0004888	5	580	140	0.60	0.64	0.62	0.43	0.48	0.45	0.24	0.29	0.26
GO:0015026	5	109	20	0.46	0.54	0.50	0.45	0.49	0.47	0.21	0.29	0.24
GO:0005220	5	42	8	0.37	0.42	0.39	0.49	0.56	0.52	0.18	0.31	0.23
GO:0030594	5	546	130	0.78	0.79	0.78	0.48	0.50	0.49	0.30	0.43	0.35
GO:0000155	5	1034	250	0.81	0.84	0.82	0.42	0.46	0.44	0.39	0.42	0.40
GO:0009881	5	289	70	0.61	0.66	0.55	0.45	0.48	0.46	0.29	0.37	0.33
GO:0008329	5	136	30	0.50	0.55	0.52	0.43	0.46	0.44	0.15	0.30	0.20
GO:0004887	5	81	20	0.44	0.53	0.48	0.46	0.59	0.52	0.09	0.16	0.12
GO:0003707	5	878	220	0.59	0.68	0.63	0.38	0.41	0.39	0.23	0.32	0.27
GO:0004896	6	130	35	0.52	0.52	0.52	0.45	0.48	0.46	0.26	0.32	0.29
GO:0016502	6	169	45	0.56	0.57	0.56	0.47	0.49	0.48	0.25	0.31	0.28
GO:0005035	6	51	10	0.46	0.48	0.47	0.51	0.53	0.52	0.00	0.00	0.00
GO:0016917	6	198	50	0.55	0.63	0.59	0.46	0.48	0.47	0.30	0.38	0.34
GO:0008066	6	301	80	0.56	0.63	0.59	0.43	0.46	0.44	0.32	0.39	0.35
GO:0008158	6	138	35	0.49	0.56	0.52	0.46	0.51	0.48	0.25	0.35	0.29
GO:0008046	6	58	15	0.44	0.45	0.44	0.47	0.55	0.51	0.00	0.00	0.00
GO:0004984	6	3474	870	0.84	0.87	0.85	0.33	0.39	0.35	0.33	0.41	0.37
GO:0035586	6	207	55	0.54	0.65	0.59	0.44	0.46	0.45	0.31	0.40	0.35
GO:0017154	6	82	20	0.51	0.59	0.56	0.47	0.56	0.51	0.16	0.20	0.18
GO:0019199	6	756	190	0.56	0.60	0.58	0.37	0.41	0.55	0.28	0.40	0.33
GO:0042813	6	141	40	0.48	0.53	0.50	0.43	0.46	0.44	0.29	0.32	0.30
GO:0004915	7	111	30	0.44	0.47	0.45	0.38	0.43	0.40	0.19	0.27	0.22
GO:0004908	7	35	10	0.38	0.39	0.38	0.54	0.57	0.55	0.17	0.29	0.21
GO:0004950	7	210	50	0.55	0.58	0.56	0.44	0.42	0.42	0.28	0.42	0.34
GO:0004897	7	29	7	0.41	0.42	0.41	0.56	0.58	0.67	0.19	0.34	0.24
GO:0004904	7	176	45	0.55	0.56	0.55	0.42	0.43	0.42	0.26	0.35	0.30

The table shows the average depth (level) of each GO term in the molecular function subontology and the accuracy of predicting the function of this term. R, P, and F DENOTE Recall, Precision, and F-value respectively

### Evaluating the performance of the three systems for predicting protein functions through 5-fold cross validation

**Fig. 1:**
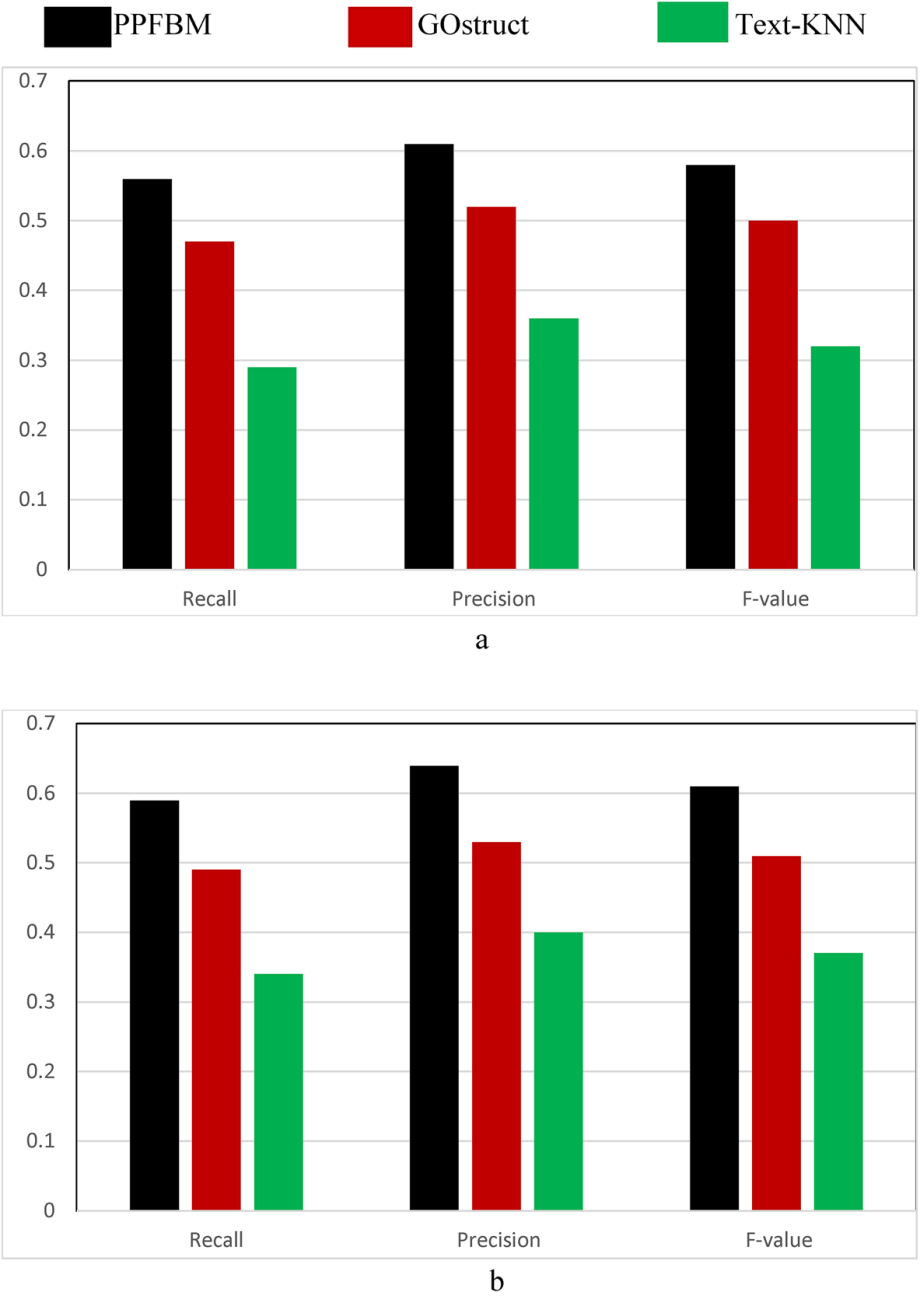
Performance of the four systems using *CAFA dataset* and 5-fold Cross Validation for predicting: (**a**) the Biological Process annotations, and (**b**) the Molecular Function annotations
- Figure 2 show the results of the complete *Saccharomyces Genome Dataset* (SGD) described previously. That is, Fig. 2 show the results of the experiments using the 6086 Yeast proteins and 46,227 PubMed abstracts associated with them according to their entries in UniProtKB database. Table 6 shows a sample of the 6086 proteins and their Biological Process annotations identified by PPFBM. The last column in the Table shows the missing annotations identified by PPFBM. We discovered that 63 % of the proteins have missing annotations based on their published annotations in GO website [51] and UniProtKB/Swiss-Prot database [28]. The following are the number of correct predictions made by each system: PPFBM: 3955, GOstruct: 3,226, and Text-KNN: 2252.

We performed 5-fold cross-validation using the three datasets described previously. Each of the three datasets is divided into five partitions at random (i.e., 5 disjoint subsets). The systems are evaluated through five runs, where in each run a different partition of the dataset is used for testing while the other four partitions are used for training the systems. Each partition is one of the five disjoint subsets of proteins and the PubMed abstracts associated with these proteins. We considered the test proteins as un-annotated, and we measured the Recall, Precision, and F-value of the systems for predicting the functions of these test proteins. As shown in Eq. 6, Z**-**score is used for determining the significance of occurrence frequency of a test protein in each set of PubMed abstracts associated with training proteins annotated with the same functional category. In the experiments, we considered a frequency of occurrences significant, if its Z-Score is above the threshold “–1.96” standard deviation. The results are shown as follows:

- Figure 1 show the results of the *CAFA dataset* [24, 50] described previously. That is, Fig. 1 show the results of the experiments using the 26,643 proteins and 94,846 PubMed abstracts associated with them according to their entries in UniProtKB database. The following are the number of correct predictions made by each system: PPFBM: 15,187, GOstruct: 12,789, and Text-KNN: 8261.

**Fig. 2:**
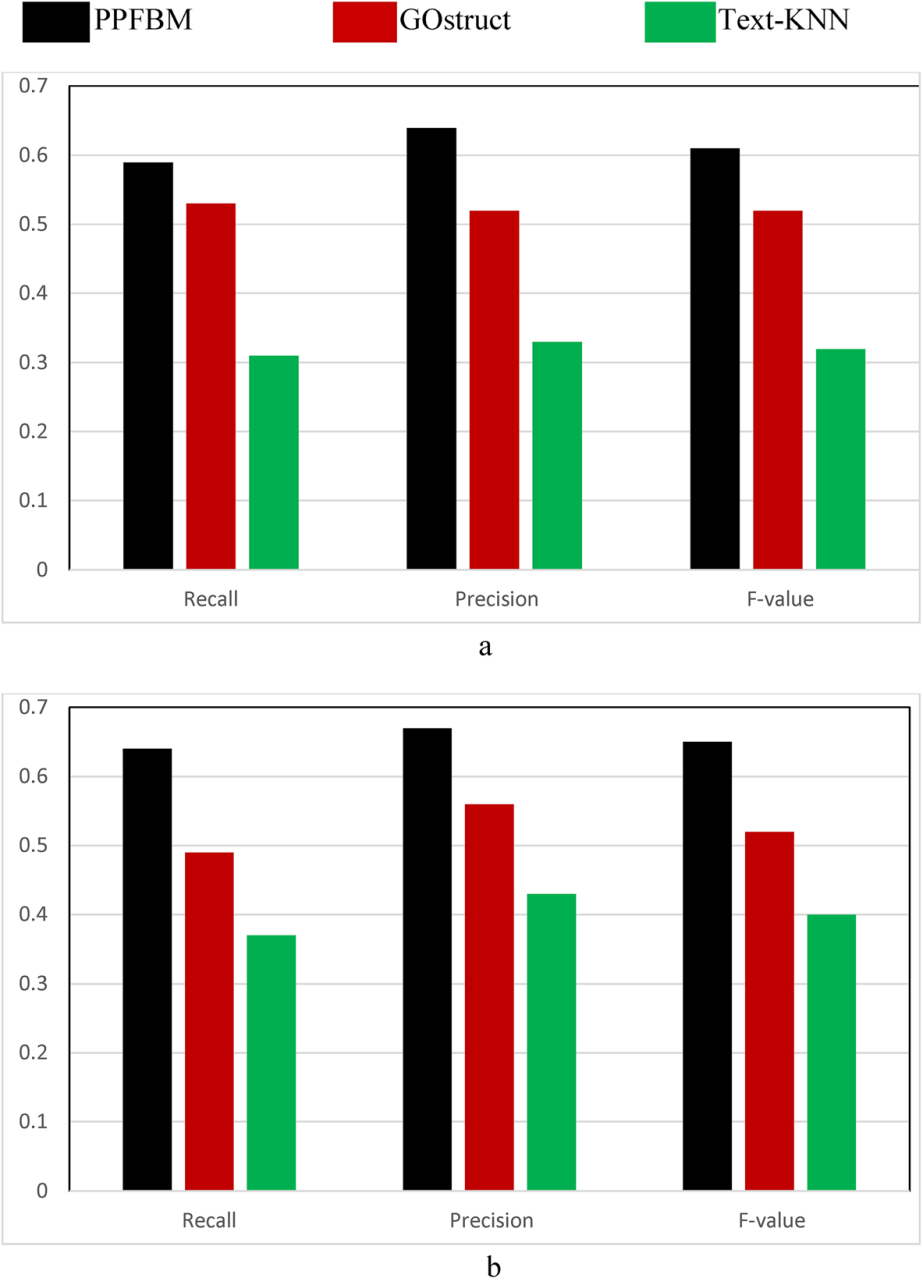
Performance of the four systems using the *Yeast protein dataset* and 5-fold Cross Validation for predicting: (**a**) the Biological Process annotations, and (**b**) the Molecular Function annotations

**Table 8:** Sample of the 6086 yeast proteins downloaded from [34] and their biological process annotations identified by PPFBM

Protein	Already published biological process annotations that are also identified by PPFBM	Missing (unpublished) annotations identified by PPFBM
YKR087C	GO:0006515 (misfolded or incompletely synthesized protein catabolic process); GO:0006508 (proteolysis)	GO:0044257 (cellular protein catabolic process)
YML120C	GO:0006120 (mitochondrial electron transport, NADH to ubiquinone); GO:0001300 (chronological cell aging); GO:0055114 (oxidation-reduction process); GO:0006116 (NADH oxidation)	GO:0042775 (mitochondrial ATP synthesis coupled electron transport); GO:0022904 (respiratory electron transport chain); GO:0045333 (cellular respiration); GO:0022900 (electron transport chain); GO:0044237 (cellular metabolic process); GO:0009987 (cellular process)
YIL156W	GO:0006511 (ubiquitin-dependent protein breakdown); GO:0006508 (peptidolysis)	GO:0044257 (cellular protein breakdown)
YJL207C	GO:0008104 (protein localization); GO:0006810 (transport); GO:0015031 (protein transport); GO:0042147 (retrograde transport, endosome to Golgi)	GO:0051179 (localization); GO:0051641 (cellular localization)
YML074C	GO:0000412 (histone peptidyl-prolyl isomerization); GO:0018208 (peptidyl-proline modification); GO:0006457 (protein folding)	GO:0000413 (protein peptidyl-prolyl isomerization)
YIL115C	GO:0031081 (nuclear pore distribution); GO:0006810 (transport); GO:0015031 (protein transport); GO:0006611 (protein export from nucleus); GO:0006607 (NLS-bearing protein import into nucleus); GO:0051028 (mRNA transport); GO:0016973 (poly(A)+ mRNA export from nucleus); GO:0000055 (ribosomal large subunit export, nucleus); GO:0000056 (ribosomal small subunit export, nucleus)	GO:0051179 (localization); GO:0034613 (cellular protein localization); GO:0008104 (protein localization); GO:0051641 (cellular localization); GO:0034504 (protein localization to nucleus); GO:0006403 (RNA localization); GO:0033750 (ribosome localization); GO:0051640 (organelle localization)
YNL305C	GO:0019722 (calcium-mediated signaling); GO:0006915 (apoptotic process); GO:0030968 (endoplasmic reticulum unfolded response)	GO:0023052 (signaling); GO:0007154 (cell communication)
YFL016C	GO:0006515 (misfolded or incompletely synthesized protein catabolic process); GO:0006457 (protein folding); GO:0006458 ('de novo' protein folding); GO:0042026 (protein refolding); GO:0006950 (response to stress); GO:0009408 (response to heat)	GO:0044257 (cellular protein catabolic process)
YGL001C	GO:0055114 (oxidation-reduction process); GO:0006694 (steroid biosynthetic process); GO:0016126 (sterol biosynthetic process); GO:0006696 (ergosterol biosynthetic process)	GO:0008610 (lipid biosynthetic process)
YJR068W	GO:0006260 (DNA replication); GO:0006298 (mismatch repair); GO:0006272 (leading strand elongation); GO:0007049 (cell cycle); GO:0007062 (sister chromatid cohesion)	GO:0006261 (DNA-dependent DNA replication); GO:0007059 (chromosome segregation); GO:0009987 (cellular process)
YOR201C	GO:0032259 (methylation); GO:0001510 (RNA methylation); GO:0006396 (RNA processing); GO:0000154 (rRNA modification)	GO:0010467 (rRNA modification); GO:0043170 (macromolecule metabolic)
YNL267W	GO:0046854 (phosphatidylinositol phosphorylation); GO:0016310 (phosphorylation); GO:0048015 (phosphatidylinositol-mediated)	GO:0007154 (cell communication); GO:0023052 (signaling)
YPR188C	GO:0007049 (cell cycle); GO:0051301 (cell division); GO:0000916 (actomyosin contractile ring contraction)	GO:0033205 (cell cycle cytokinesis); GO:0000910 (cytokinesis); GO:0022402 (cell cycle process); GO:0009987 (cellular process)
YOR332W	GO:0007035 (vacuolar acidification); GO:0015991 (ATP hydrolysis coupled proton transport); GO:0006810 (transport); GO:0006811 (ion transport); GO:0015992 (proton trans)	GO:0051179 (localization)
YJR042W	GO:0006606 (protein import into nucleus); GO:0000055 (ribosomal large subunit export from nucleus); GO:0051028 (mRNA transport); GO:0006406 (mRNA transport); GO:0006810 (transport); GO:0015031 (protein transport); GO:0031081 (nuclear pore distribution)	GO:0034504 (protein localization to nucleus); GO:0006403 (RNA localization); GO:0033365 (protein localization to organelle); GO:0008104 (protein localization); GO:0051641 (cell. localization); GO:0033036 (macromolecule localization); GO:0051179 (localization); GO:0033750 (ribosome localization)
YNL090W	GO:0007017 (microtubule-based process); GO:0030010 (establishment of cell polarity); GO:0007015 (actin filament organization); GO:0007264 (small GTPase mediated signal transduction)	GO:0007154 (cell communication); GO:0023052 (signaling)
YMR223W	GO:0006511 (ubiquitin-dependent protein catabolic process); GO:0006351 (transcription, DNA-templated); GO:0034729 (histone H3-K methylation); GO:0051568 (histone H3-K4 methylation); GO:0006508 (proteolysis); GO:0016578 (histone deubiquitination)	GO:0044257 (cell protein catabolic process); GO:0043170 (macromolecule metabolic process); GO:0008152 (metabolic proc.); GO:0010467 (gene exp.)
YML085C	GO:0006184 (GTP catabolic process); GO:0007017 (microtubule-based process); GO:0000070 (mitotic sister chromatid segregation); GO:0045143 (homologous chromosome segregation); GO:0030473 (nuclear migration along microtubule)	GO:0051647 (nucleus localization); GO:0000747 (conjugation with cellular fusion); GO:0051640 (organelle localization); GO:0051641 (cellular localization); GO:0000746 (conjugation); GO:0051704 (multi-organism process); GO:0007018 (microtubule-based movement); GO:0022403
YPR187W	GO:0006351 (transcription, DNA-templated); GO:0006360 (transcription from RNA polymerase I promoter); GO:0006366 (transcription from RNA polymerase II promoter); GO:0006383 (transcription from RNA polymerase III promoter); GO:0042797 (tRNA transcription from RNA polymerase III promoter)	GO:0043170 (macromolecule metabolic process); GO:0008152 (metabolic process); GO:0010467 (gene expression)
YGL103W	GO:0006412 (translation); GO:0002181 (cytoplasmic translation); GO:0046677 (response to antibiotic); GO:0046898 (response to cycloheximide)	GO:0010467 (gene expression); GO:0043170 (macromolecule metabolic process); GO:0008152 (metabolic process)
YGR216C	GO:0006506 (GPI anchor biosynthetic process)	GO:0042158 (lipoprotein biosynthetic process)
YER157W	GO:0016236 (macroautophagy); GO:0030242 (peroxisome degradation); GO:0006886 (intracellular protein transport); GO:0006810 (transport); GO:0015031 (protein transport); GO:0032258 (CVT pathway); GO:0006888 (ER Golgi vesicle-mediated transport); GO:0006891 (intra-Golgi vesicle-mediated transport); GO:0000301 (retrograde transport within Golgi)	GO:0008104 (protein localization); GO:0051641 (cellular localization); GO:0033036 (macromolecule localization); O:0051179 (localization); GO:0034613 (cellular protein localization)
YGR247W	GO:0009187 (cyclic nucleotide metabolic process)	GO:0016070 (RNA metabolic process)
YGL243W	GO:0006396 (RNA processing); GO:0006400 (tRNA modification); GO:0008033 (tRNA processing)	GO:0010467 (gene expression); GO:0043170 (macromolecule metabolic process); GO:0008152 (metabolic process)
YMR166C	GO:0055085 (transmembrane transport); GO:0006810 (transport)	GO:0051179 (localization)
YMR178W	GO:0008150 (biological_process); GO:0006777 (Mo-molybdopterin cofactor biosynthetic process)	GO:0044267 (cellular protein metabolic process)
YML077W	GO:0006914 (autophagy); GO:0006810 (transport); GO:0016192 (vesicle-mediated transport); GO:0006888 (ER vesicle- transport)	GO:0051179 (localization); GO:0051641 (cellular localization)
YML073C	GO:0006412 (translation); GO:0002181 (cytoplasmic translation)	GO:0043170 (macromolecule metabolic proc.); GO:0008152 (metabolic proc.)
YOR035C	GO:0007533 (mating type switching); GO:0030036 (actin cytoskeleton organization); GO:0008298 (intracellular mRNA localization)	GO:0030154 (cell differentiation); GO:0032505 (reproduction of a single-celled organism); GO:0000003 (reproduction)
YOR222W	GO:0055085 (transmembrane transport); GO:0006810 (transport); GO:0006839 (mitochondrial transport)	GO:0051179 (localization); GO:0051641 (cellular localization)
YNL135C	GO:0018208 (peptidyl-proline modification); GO:0000413 (protein peptidyl-prolyl isomerization); GO:0006457 (protein folding)	GO:0009092 (homoserine metabolic process)
YGL200C	GO:0006810 (transport); GO:0015031 (protein transport); GO:0016192 (vesicle-mediated transport); GO:0006888 (ER to Golgi vesicle-mediated transport)	GO:0051179 (localization); GO:0051641 (cellular localization)
YGR260W	GO:0055085 (transmembrane transport); GO:0006810 (transport); GO:0015890 (nicotinamide mononucleotide transport)	GO:0051179 (localization)
YPR166C	GO:0006412 (translation); GO:0032543 (mitochondrial translation)	GO:0010467 (gene expression); GO:0043170 (macromolecule metabolic proc.)
YKR019C	GO:0006914 (autophagy); GO:0006629 (lipid metabolic process); GO:0009267 (cellular response to starvation); GO:0000183 (chromatin silencing at rDNA); GO:0048017 (inositol lipid-mediated signaling); GO:0032258 (CVT pathway)	GO:0007154 (cell communication); GO:0023052 (signaling); GO:0034613 (cellular protein localization); GO:0008104 (protein localization); GO:0051641 (cellular localization); GO:0051179 (localization)
YLR348C	GO:0006810 (transport); GO:0006817 (phosphate ion transport)	GO:0051179 (localization)
YLR431C	GO:0006914 (autophagy); GO:0034497 (protein localization to pre-autophagosomal structure); GO:0006810 (transport); GO:0015031 (protein transport); GO:0032258 (CVT pathway)	GO:0034613 (cellular protein localization); GO:0008104 (protein localization); GO:0051179 (localization)
YJL004C	GO:0006810 (transport); GO:0015031 (protein transport); GO:0043001 (Golgi to plasma membrane protein transport); GO:0006895 (Golgi to endosome transport)	GO:0051179 (localization); GO:0034613 (cell protein localization); GO:0008104 (protein localization); GO:0051641 (cell localization)
YFL055W	GO:0055085 (transmembrane transport); GO:0003333 (amino acid transmembrane transport); GO:0006810 (transp)	GO:0051179 (localization)
YPR179C	GO:0006351 (transcription, DNA-templated); GO:0016575 (histone deacetylation); GO:0007059 (chromosome segregation); GO:0010978 (gene silencing involved in chronological cell aging); GO:0031047 (gene silencing by RNA)	GO:0043170 (macromolecule metabolic process); GO:0008152 (metabolic process); GO:0001300 (chronological cell aging); GO:0007568 (aging); GO:0009987 (cellular process)

**Fig. 3:**
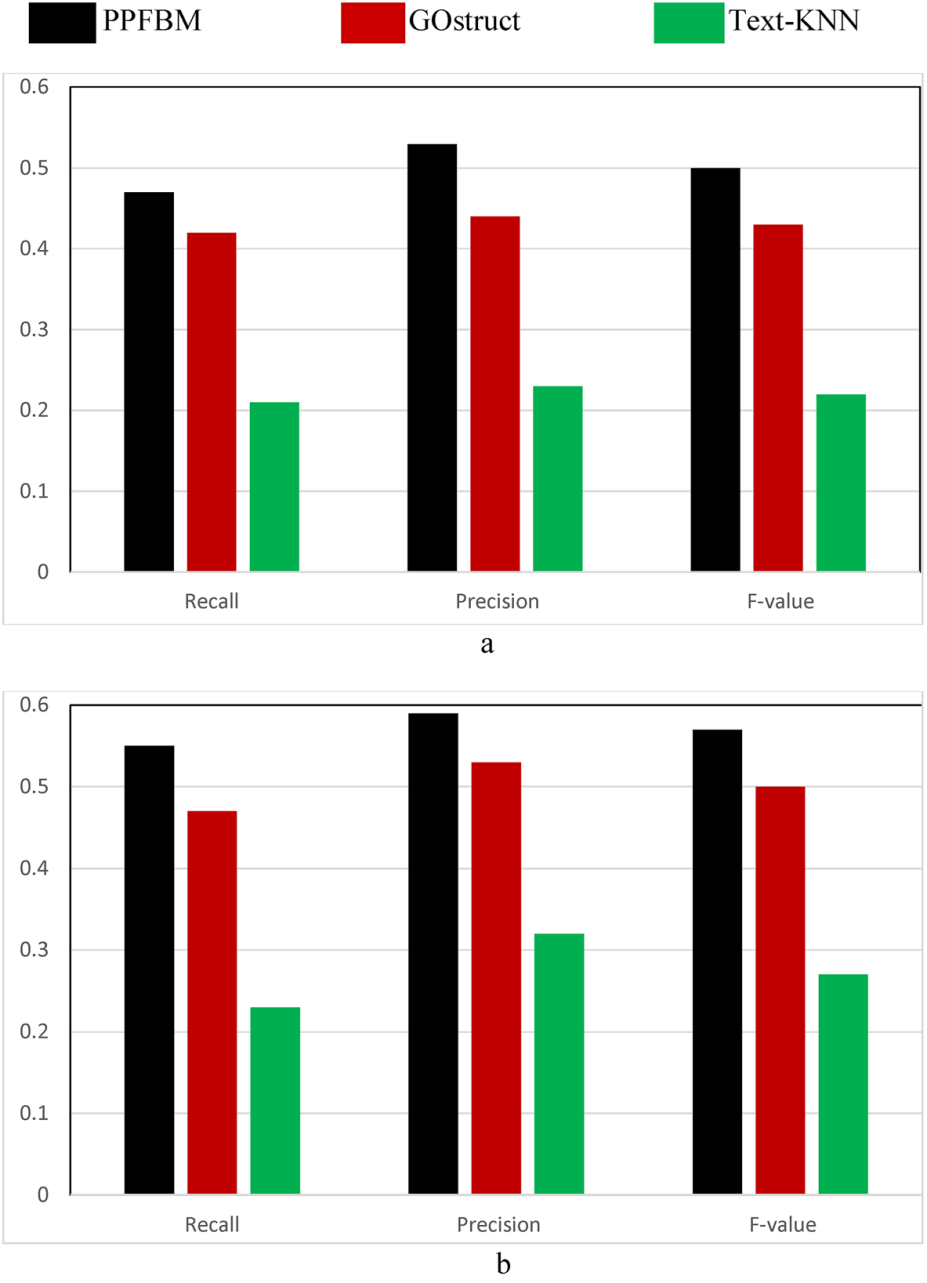
Performance of the four systems using the *GO dataset* and 5-fold Cross Validation for predicting: (**a**) the Biological Process annotations, and (**b**) the Molecular Function annotations

The already known annotations and also the missing annotations Identified by PPFBM are both shown. A demo of PPFBM that identifies the biological process annotations of the complete yeast protein dataset is available at: http://ecesrvr.kustar.ac.ae:8080/PPFBM/
- Figure 3 show the results of the *Gene Ontology (GO) dataset* described previously. That is, Fig. 3 show the results of the experiments using the 78,962 proteins and the 577,486 PubMed abstracts associated with them according to their entries in UniProtKB. The following are the number of correct predictions made by each system: PPFBM: 41,060, GOstruct: 33,953, and Text-KNN: 17,372.

**Fig. 4:**
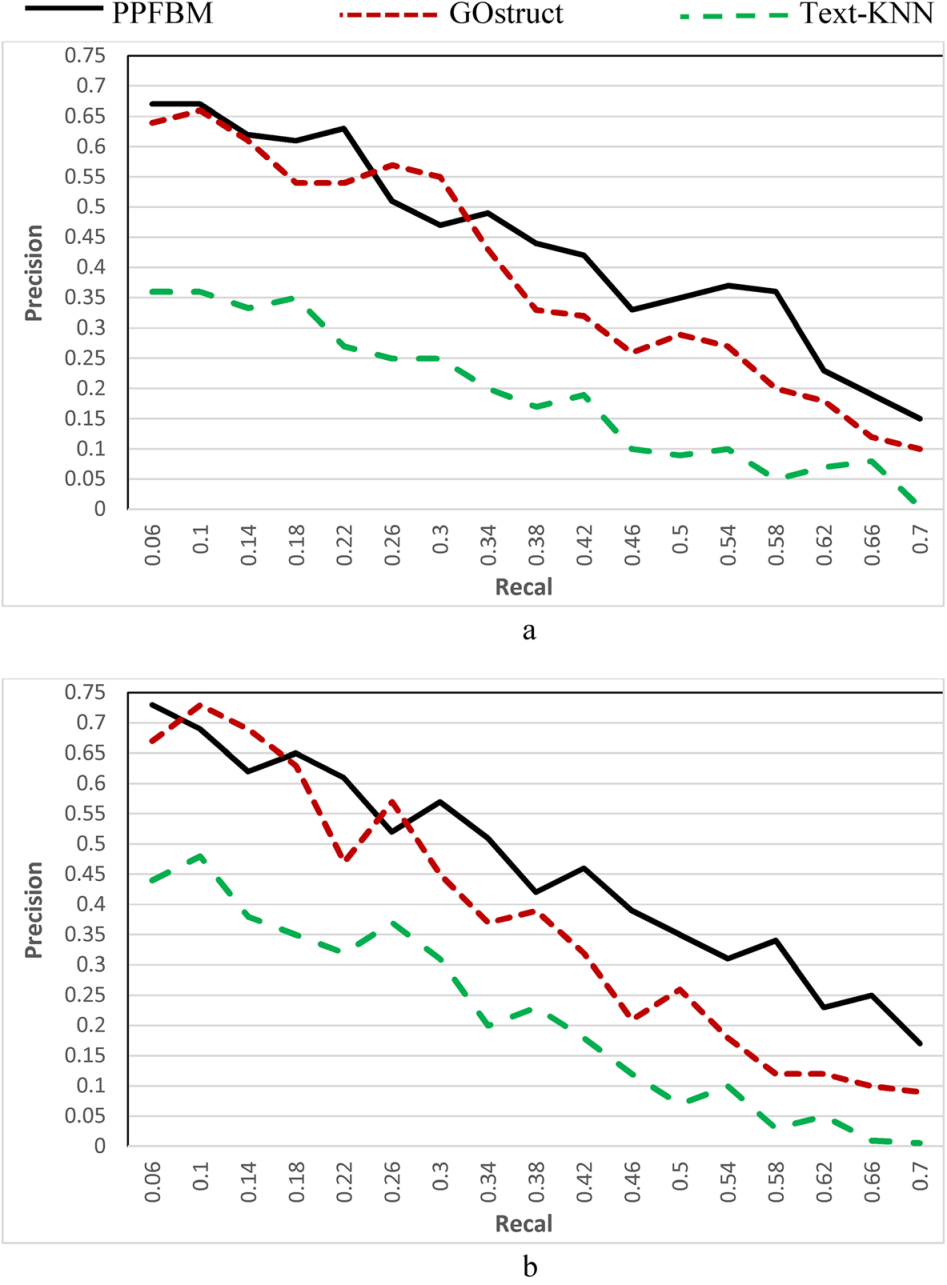
Precision-Recall curves plotted using *CAFA protein-centric metrics* with confidence scores above thresholds distributed evenly in the range [0, 1] at step size 0.01. (**a**) shows the curves for the Biological Process annotations, and (**b**) shows the curves for the Molecular Function annotations

As shown in Fig. 4, we also evaluated the three systems using *CAFA protein-centric metrics*. We followed CAFA [24, 50] procedure for plotting precision-recall curve according to a sliding threshold scheme. Only predictions with confidence scores higher than threshold values *t* (0 < = *t* < = 1) are selected for the evaluation. We used thresholds distributed evenly in the range [0, 1] at step size 0.01. At each threshold, we calculated the precision and recall for each protein and also the average precision and recall on all the protein dataset. At each threshold *t*, the Recall rc_i_ (*t*) and Precision pr_i_(*t*) for each protein *i* are calculated as shown in Eqs. 7 and 8:

7 $$ p{r}_i(t)=\frac{{\displaystyle {\sum}_fI\kern0.1em \left(f\in {P}_i(t)\wedge f\in {T}_i\right)}}{{\displaystyle {\sum}_fI\kern0.1em \left(f\in {P}_i(t)\right)}} $$

8 $$ r{c}_i(t)=\frac{{\displaystyle {\sum}_fI\kern0.1em \left(f\in {P}_i(t)\wedge f\in {T}_i\right)}}{{\displaystyle {\sum}_fI\kern0.1em \left(f\in \kern0.3em {T}_i\right)}} $$

where: (1) *Ti* is the set of functional categories that is experimentally determined for protein *i*, (2) *Pi*(*t*) is the set of functional categories predicted by a system for protein *i* with score greater than or equal to *t,* (3) *f* is a functional term in the ontology, and (4) *I*(·) is the standard indicator function. The overall Recall and Precision for protein *i* at threshold *t* are calculated as shown in Eqs 9 and 10.

9 $$ pr(t)=\frac{1}{m(t)}.{\displaystyle \sum_{i=1}^{m(t)}p{r}_i(t)} $$

10 $$ rc(t)=\frac{1}{n}.{\displaystyle \sum_{i=1}^nr{c}_i(t)} $$

where *m*(*t*) is the number of proteins that have at least one prediction above *t* and *n* is the number of all proteins in the dataset. Figure 4 show the results.

**Fig. 5:**
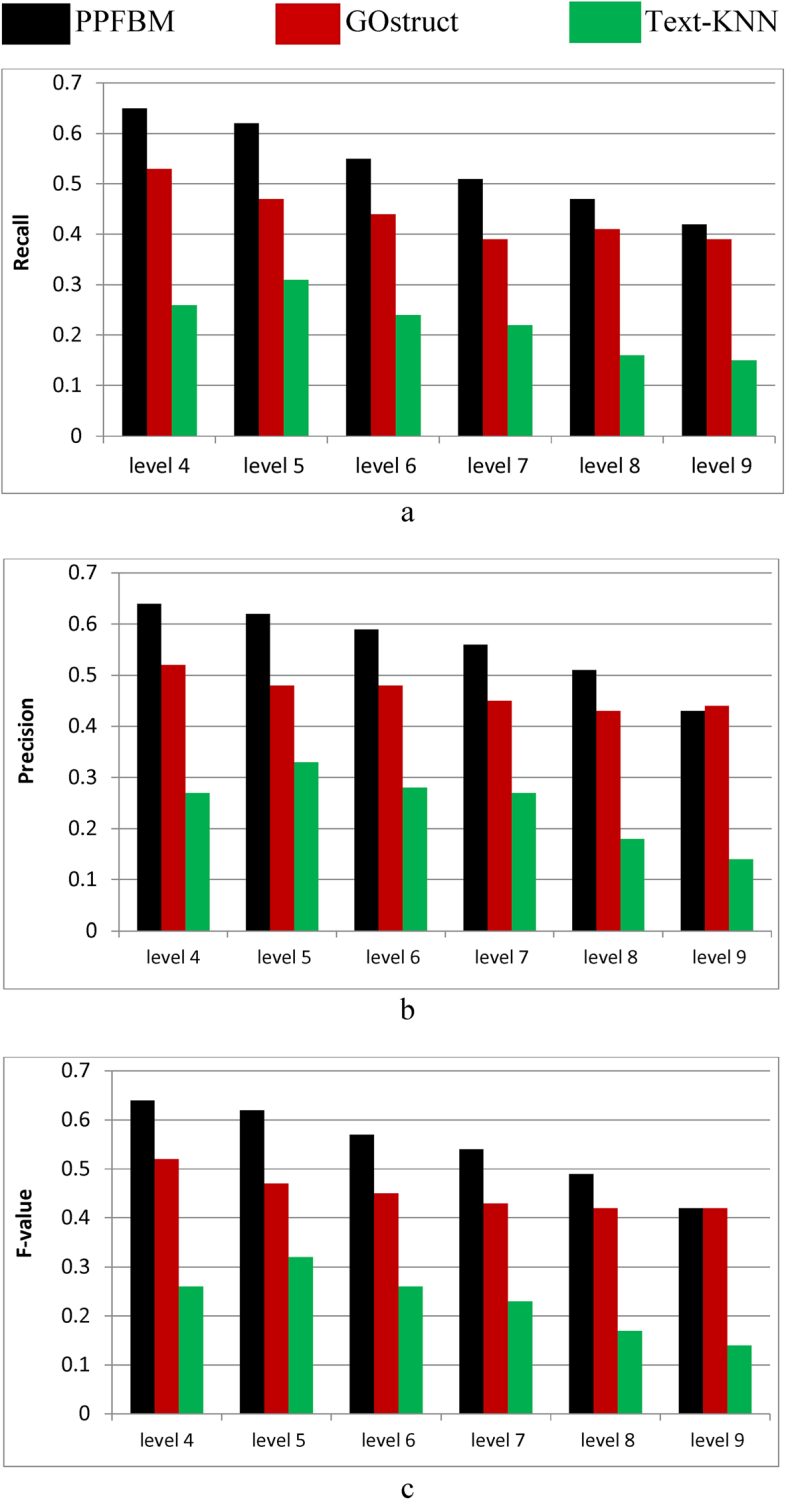
The average Recall, Precision, and F-value of predicting the functions of each set of GO terms located at the same average depth (level) in the Biological Process subontology

**Fig. 6:**
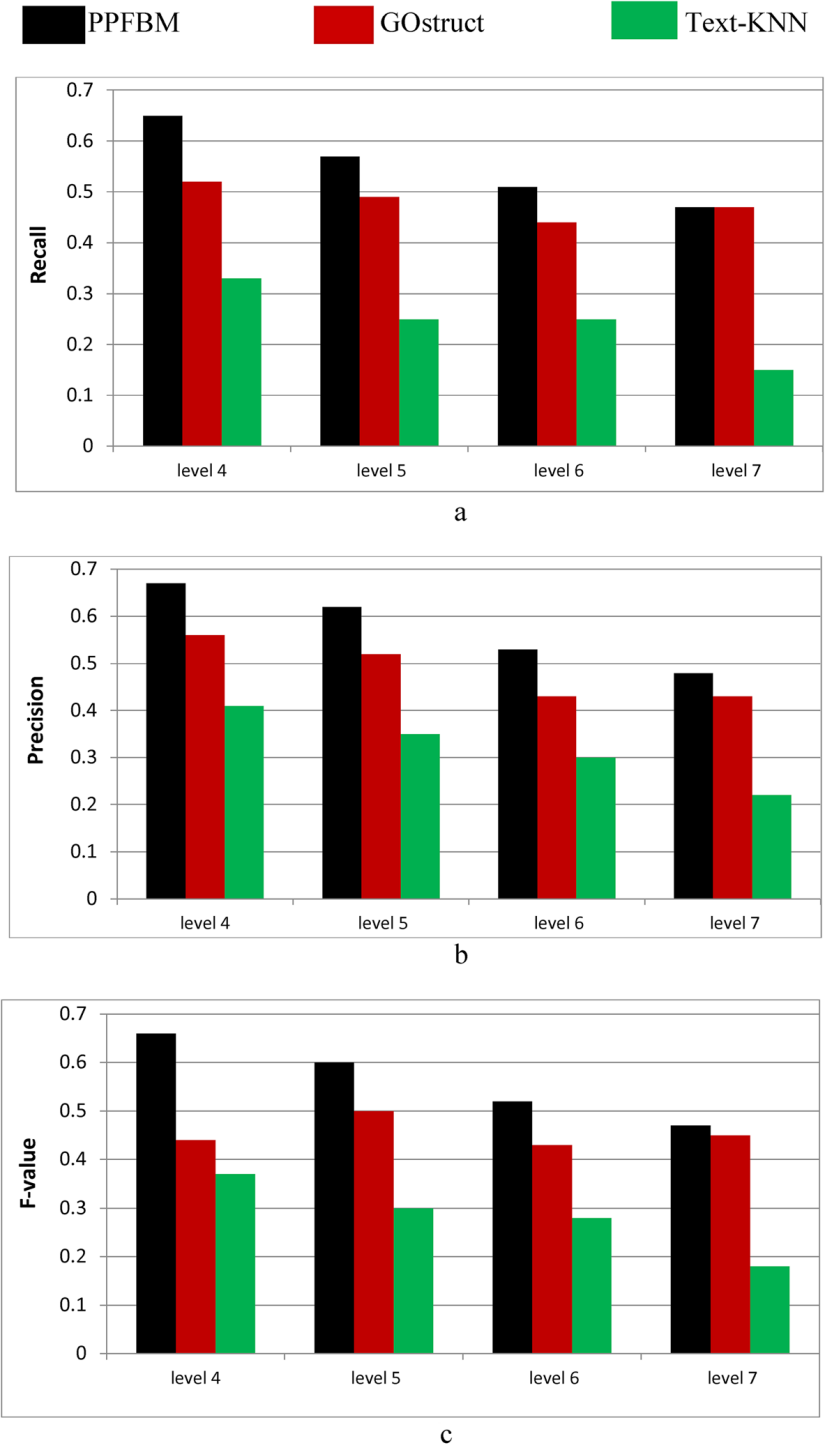
The average Recall, Precision, and F-value of predicting the functions of each set of GO terms located at the same average depth (level) in the Molecular Function subontology

We also measured the Recall, Precision, and F-value of the systems for predicting the function of each GO term. For each GO term *t*, we randomly selected a set of training proteins and a set of testing proteins annotated with the function of *t*. We evaluated the accuracy of the systems for predicting the function of *t*. The results are shown as follows. Figure 5 shows the accuracy of predicting the functions of each set of GO terms located at the same average depth (level) in the Biological Process ontology. Figure 6 shows the accuracy of predicting the functions of each set of GO terms located at the same depth (level) in the Molecular Function ontology. Tables 7 and 8, show the depth (level) of each GO term in GO Graph and the accuracy of predicting the function of this term.

### Evaluating the performance of the three systems for predicting protein functions through cumulative-validation

**Fig. 7:**
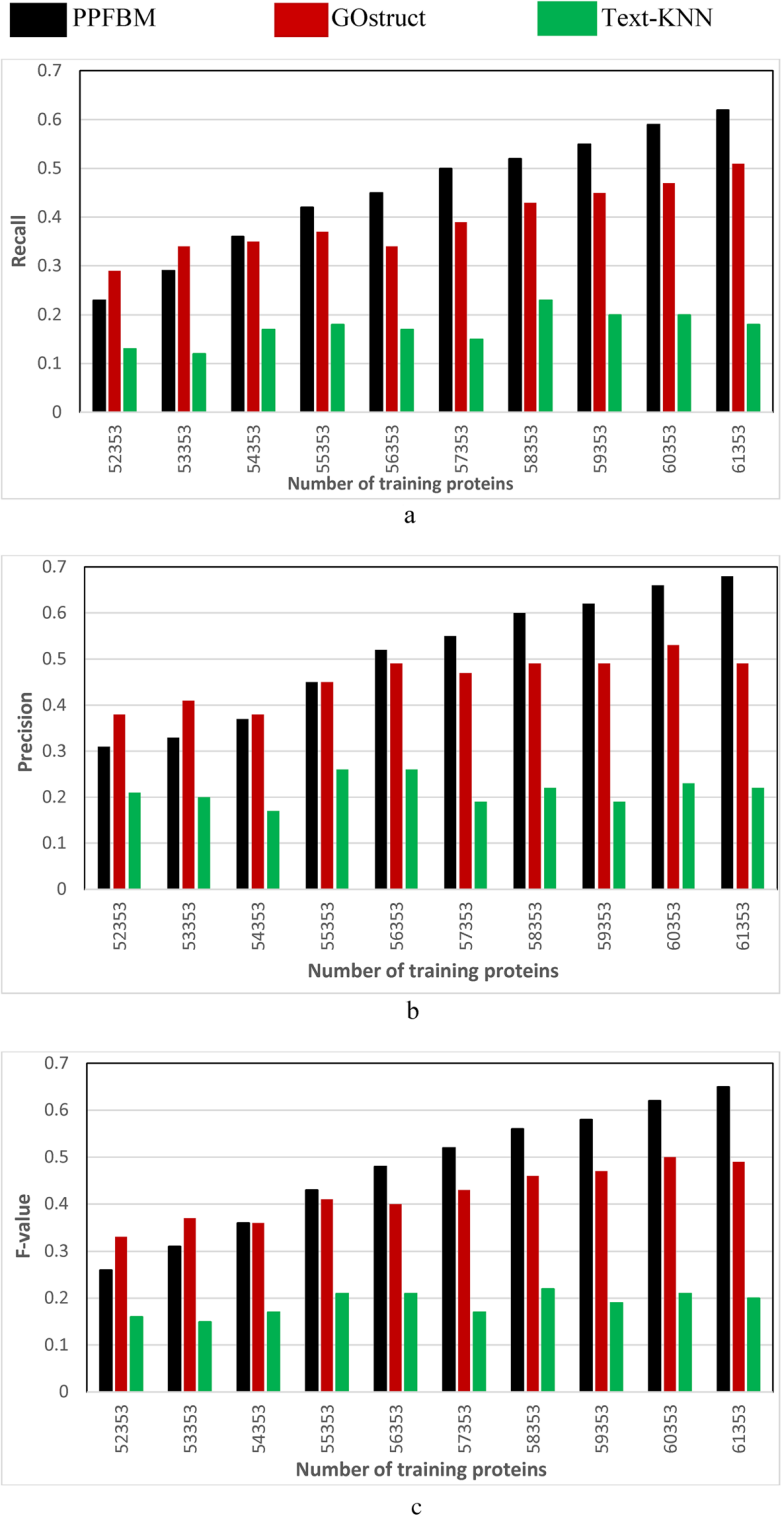
The *Recall*, *Precision,* and *F-value* for predicting *GO Biological Process annotations* using a successively accumulating set of training proteins

**Fig. 8:**
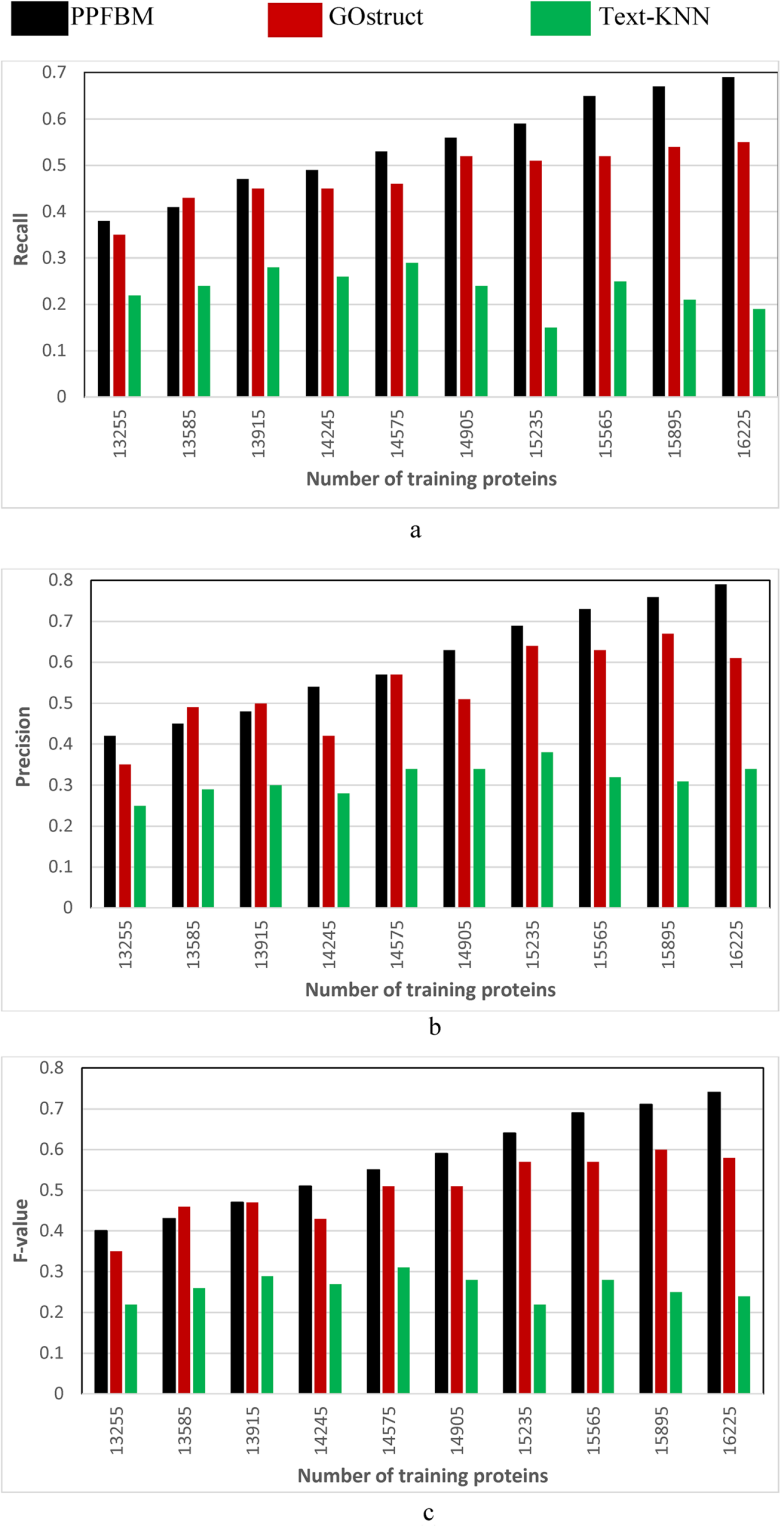
The *Recall*, *Precision,* and *F-value* for predicting *GO Molecular Function annotations* using a successively accumulating set of training proteins

In this test, we perform ten runs using the GO dataset described previously. The number of training proteins accumulates successively in each run. In each run, 1330 test proteins *(*i.e.*, 1000 test proteins from the Biological Process subontology and 330 test proteins from the Molecular Function subontology)* are considered un-annotated and their functions are determined based on the current set of training proteins. The first run was performed using: (1) 52,353 training proteins from the Biological Process subontology and 13,255 proteins from the Molecular Function subontology, and (2) 1000 test proteins from the Biological Process subontology and 330 test proteins from the Molecular Function subontology. The set of training proteins in each of the nine subsequent runs consists of the set of training proteins used in the predecessor run in addition to the 1330 test proteins used in the predecessor run (i.e., 1000 test proteins from the Biological Process subontology and 330 test proteins from the Molecular Function subontology). That is, the number of training proteins accumulates successively in each run by adding the 1330 test proteins used in the predecessor run to the current set of training proteins. Figures 7 and 8 show the performance of each system in each of the ten runs.

### Discussion of the results

#### The impact of the Key concepts employed by PPFBM on prediction results

As Figs. 1, 2, 3, 4, 5, 6, 7 and 8 show, PPFBM outperformed GOstruct and Text-KNN. We attribute the performance of PPFBM over the other two systems to the following factors:

1. The first factor is the employment of PPFBM to the concept of *dominant molecules* to represent proteins. This concept ensures that uninformative molecules are filtered and excluded from representing proteins. A molecule is considered uninformative if it has only few occurrences in abstracts and/or is assigned a high weight even though it is found in abstracts associated with many other protein classes. The poor performance of Text-KNN is attributed, mainly, to the fact that it does not employ a mechanism for filtering and excluding *uninformative characteristic terms* from representing proteins.
2. The second factor is the employment of PPFBM to the concept of *semantic relationship* between proteins and molecules in sentences. This concept ensures each co-occurrence of a molecule and protein pair in a sentence is disregarded, if the pair is unrelated grammatically (as described previously). That is, PPFBM considers the co-occurrence of a molecule and protein pair in a sentence as an indicative of their association only if the pair is semantically related. GOstruct and Text-KNN *do not* consider the concept of semantic relationship. For example, Text-KNN considers the occurrences of a term *t* in an abstract associated with a protein *p*_*1*_ as indicative of the association between *t* and *p*_*1*_ (if *t* passes the Z-Score threshold), while it overlooks the contexts in which *t* occurs. The term *t* may be associated with a protein other than *p*_*1*_, even though it occurs within an abstract(s) associated with *p*_*1*_. Consider for example that *t* and a protein *p*_*2*_ are semantically related based on their co-occurrences in the sentences of an abstract(s) associated with *p*_*1*_. In this case, *t* is likely to be associated with *p*_*2*_ and it may not necessary be associated with *p*_*1*_ even though it occurs in an abstract(s) associated with *p*_*1.*_ Thus, the occurrences of *t* in an abstract associated with *p*_*1*_ may not always be an indicative of the association between *t* and *p*_*1*_. We cannot determine this without checking the contexts in which terms occur within sentences (e.g., checking the semantic relationships between terms in sentences).

### The impact of the size of GO annotation terms on prediction results

We analysed the results of the experiments conducted using GO dataset. We observed from the results of predicting the functions of individual GO terms the following. As the number of training proteins annotated with the function of a term *T* gets larger, PPFBM tends to predict the function of *T* more accurately. This can be seen in the results shown in Tables 6 and 7. This is because, as the number of training proteins gets larger, PPFBM computes the beats/looses scores of molecules more accurately (recall Table 4). PPFBM may not predict the functions of very small classes accurately (classes with fewer than about 100 training proteins). On the other hand, GOstruct tends to predict more accurately the functions of GO terms annotating very small number of training proteins. This is attributed to the fact that GOstruct orders functions based on their influences and gives higher influences to functions with smaller number of proteins annotated with them. This is disadvantageous to GOstruct, since the size of training proteins gets larger over time as un-annotated proteins are assigned functions. As for PPFBM, as the set of training proteins annotated with the function of a GO annotation term *T* gets larger, the set of dominant molecules representing *T* becomes more optimized and more accurate. This is because the larger the number of training proteins gets, the more accurate becomes the scores assigned to molecules based on their number of beats and looses (recall Table 4).

### The impact of the size of training proteins on prediction results

As Figs. 7 and 8 show, PPFBM’s performance over the other two systems increases steadily as the number of training protein increases. That is, PPFBM’s prediction performance becomes more accurate constantly, as the size of training proteins gets larger. This is because every time a new set of test proteins is added to the current set of training proteins, PPFBM optimizes its prediction performance as follows:

1. It updates and optimizes the set of dominant molecules representing each training protein *p* in the current set of training proteins. It does so by updating the beats/looses scores and normalized weights (recall Table **4**) of the molecules associated with *p* based on the occurrences of these molecules in the abstracts associated with the test proteins that have recently been added to the current set of training proteins.
2. It optimizes the computation of the significance of occurrence frequency of the set *S*_*r*_ of proteins that is semantically similar to an un-annotated protein in PubMed abstracts (as described previously). It does so by updating the number of PubMed abstracts associated with each functional category *FC* by adding the abstracts associated with the test proteins annotated to *FC* that have recently been added to the current set of training proteins. This improves the computation of Z-score (recall Eq. **6**), which improves the prediction performance of PPFBM. As a result, the accuracy of predicting the functional category *FC* as the functional category of succeeding un-annotated proteins (e.g., in the coming runs) improves.

Thus, PPFBM’s prediction performance improves over time as each previously un-annotated set of protein is assigned functional categories and is associated with abstracts in biomedical databases. As for GOstruct, and Text-KNN, the increment of the size of training proteins has no significant impact on their prediction performance.

## Conclusions

We proposed in this paper an information extraction system called PPFBM that predicts the functions of un-annotated proteins. PPFBM overcomes the limitations of most current constituency and dependency parsers by employing novel NLP dependency parsing and information extraction techniques. These techniques identify the *semantic relationship* between each pair of terms in a sentence using novel semantic rules that conform to grammar and linguistics theories. PPFBM represents each protein by the other molecules that associate with it and are found within the biomedical abstracts associated with the protein. PPFBM determines the functions of un-annotated protein *p* as follows. First, it determines the set *S*_*r*_ of annotated proteins that is semantically similar to *p* by matching the dominant molecules representing *p* and the dominant molecules representing the annotated proteins. It will assign the un-annotated protein *p* the functional category *FC*, if the significance of the frequency of occurrences of set *S*_*r*_ in biomedical abstracts associated with proteins annotated with *FC* is statistically significantly different from others. We evaluated the quality of PPFBM by comparing it experimentally with GOstruct [21, 22] and Text-KNN [24] for predicting the functions of proteins. We used for the evaluation three different datasets: CAFA dataset [24, 50], Saccharomyces Genome Dataset (SGD) [36], and a subset of Gene Ontology (GO) dataset [51]. We performed 5-fold cross-validation as well as Cumulative-Validation (through a successively accumulating set of training proteins) using the three datasets. Results showed that PPFBM outperformed the two systems in terms of Recall, Precision, and F-value.

We attribute the performance of PPFBM over the two systems to the following factors: (1) the employment of PPFBM to the concept of *dominant molecules* to represent proteins, (2) the employment of PPFBM to the concept of *semantic relationship* between proteins and molecules in sentences, (3) the fact that PPFBM updates and optimizes the set of dominant molecules representing each training protein *p* in the current set of training proteins, by updating the beats/looses scores of the molecules associated with *p* based on the occurrences of these molecules in the abstracts associated with the test proteins that have recently been added to the current set of training proteins, and (4) the fact that PPFBM optimizes the computation of the significance of occurrence frequency of the set of proteins that is semantically similar to an un-annotated protein in PubMed abstracts,

## Appendix

The demo of PPFBM annotates an input Yeast protein with the functions of Gene Ontology (GO) terms from the Biological Process sub-ontology using the same techniques described in this paper. The demo application represents each Yeast protein by the dominant molecules that associate with it and are found within PubMed abstracts associated with the protein. After the user enters a Yeast protein *p* and clicks the “search” button, the demo application will determine the set of Gene Ontology terms, whose functions will be used by the application to annotate *p*, as follows. First, the application will determine the set *S*_*r*_ of Yeast training proteins that is semantically similar to *p* by matching the dominant molecules representing *p* and the dominant molecules representing the training proteins. Then, the application will assign protein *p* the functions of a Gene Ontology term *t*, if the significance of the frequency of occurrences of set *S*_*r*_ in PubMed abstracts associated with Yeast proteins annotated to the functions of *t* is statistically significantly different from the significance of the frequency of occurrences of set *S*_*r*_ in PubMed abstracts associated with Yeast proteins annotated to the functions of *all other* Gene Ontology terms. The demo uses 6086 Yeast proteins downloaded from [44]. The Gene Ontology dataset was downloaded from [41]. The PubMed abstracts associated with the proteins are retrieved based on the entries of these proteins in UniProtKB/Swiss-Prot database [38].
