# Erratum to: Predicting the functions of a protein from its ability to associate with other molecules

**DOI:** 10.1186/s12859-016-0953-5

**Published:** 2016-02-26

**Authors:** Kamal Taha, Paul D. Yoo

**Affiliations:** 1 Department of Electrical and Computer Engineering, Khalifa University, Abu Dhabi, United Arab Emirates; 2 Faculty of Science and Technology, Bournemouth University, Bournemouth, UK

Upon publication of the original article [1] it was found that author corrections for Table 1 were not correctly implemented. You can now find the correct Table 1 below and also updated in the original article.

**Table 1:** The distribution of semantically related and semantically unrelated co-occurrences of molecule *m*_*i*_ and Protein *p* Pair in an Abstract *A*_*j*_

*m*_*i*_ and *p* co-occur in the same sentence	Yes	No	Total
*m*_*i*_ and *p* are semantically related			
Yes	*o*_11_	*o*_12_	*R*_*1*_
No	*o*_21_	*o*_22_	*R*_*2*_
Total	*C*_*1*_	*C*_*2*_	*N*
